# PAK2 is essential for chromosome alignment in metaphase I oocytes

**DOI:** 10.1038/s41419-023-05585-7

**Published:** 2023-02-22

**Authors:** Juan Zeng, Shiwei Wang, Min Gao, Dian Lu, Shuang Song, Diyu Chen, Weimin Fan, Zhiliang Xu, Zhiguo Zhang, Xiaofang Sun

**Affiliations:** 1 Department of Obstetrics and Gynecology, Key Laboratory for Major Obstetric Diseases of Guangdong Province, The Third Affiliated Hospital of Guangzhou Medical University, Guangzhou, Guangdong China; 2 Key Laboratory of Reproduction and Genetics of Guangdong Higher Education Institutes, Guangzhou, Guangdong China; 3 Department of Obstetrics and Gynecology, Reproductive Medicine Center, the First Affiliated Hospital of Anhui Medical University, Anhui, China; 4 College of Animal Science and Technology, China Agricultural University, Beijing, China; 5 MOE Key Laboratory of Gene Function and Regulation, State Key Laboratory of Biocontrol, School of Life Sciences, Sun Yat-sen University, Guangzhou, China

**Keywords:** Meiosis, Chromosome segregation

## Abstract

As a highly conserved and ubiquitously expressed serine/threonine kinase, p21-activated kinase 2 (PAK2) participates in diverse biologic events. However, its roles in mouse oocyte meiotic maturation remain unclear. The present study revealed that mouse oocytes depleted of *Pak2* were unable to completely progress through meiosis and that a majority were arrested at metaphase I. *Pak2* depletion thus prompted MI arrest and induced meiotic chromosome alignment defects in mouse oocytes, in part due to a reduction in polo-like kinase (PLK1). We demonstrated that PAK2’s interaction with PLK1 protected it from degradation by APC/C^Cdh1^, and that it promoted meiotic progression and bipolar spindle formation. Our data collectively display critical functions for PAK2 in meiotic progression and chromosome alignment in mouse oocytes.

## Introduction

Bipolar spindle assembly is a prerequisite for accurate chromosomal segregation and the prevention of aneuploidy [1]. Although the spindle in somatic and male germ cells is formed by microtubules that are nucleated by canonical centrosomes [2], mouse oocyte meiotic spindles are assembled in multiple, discrete microtubule organizing centers (MTOCs) without canonical centrosomes [3]. As bipolar spindles are organized in mouse oocytes in the absence of standard centrosomes [4], acentriolar MTOCs (aMTOCs) thereby serve as a major site of microtubule nucleation and functionally replace centrosomes in mouse oocytes [3]. During meiotic progression, multiple aMTOCs are fragmented into a large number of small MTOCs, redistributed toward the spindle poles, and merged into two equal spindle poles [4]. Any error in bipolar spindle formation can result in chromosome-segregation defects during meiosis and thus lead to aneuploidy [5]. Aneuploidy is a leading cause of spontaneous abortions, birth defects, and developmental disabilities in humans [6]. While numerous molecules that affect spindle/chromosome organization have been proposed for oocyte meiosis, the underlying pathways that regulate the meiotic assembly structure remain obscure.

PAKs are evolutionarily conserved serine/threonine protein kinases that play major roles in multiple biological processes, largely through their effects on cytoskeletal dynamics [7], cellular senescence [8], cytostasis [9], apoptosis [10], and angiogenesis [11]. The PAK family thus far consists of six members and is divided into group I PAKs (PAKs 1–3) and group II PAKs (PAKs 4–6) [12]. In mice, *Pak1*-KO and *Pak3*-KO are viable, healthy, and fertile ^13^; but *Pak2*-KO and endothelial depletion of *Pak2* lead to early embryonic lethality [13]. In vivo, *Pak2* cardiac-deleted mice (*Pak2*-CKO) manifested endoplasmic reticulum stress, cardiac dysfunction, and severe cell death [14]. In vitro, *Pak2* depletion in adult endothelial cells leads to severe apoptosis and acute angiogenic defects [15]. In addition, *Pak2* deficiency impaired actin-cytoskeleton remodeling [7], and *Pak2* haploid deficiency resulted in synaptic cytoskeletal damage [16]. Although the function of PAK2 in oocyte meiosis is unknown, the gain or loss of PAK function directly affects polo-like kinase (PLK1) [17].

PLK1 has been reported to be essential for centrosomal maturation, bipolar spindle assembly, and chromosome segregation in mammalian mitosis and meiosis [18, 19]. In mitosis, PLK1 is expressed at a very low level at the G1/S transition, and its expression increases during the S phase and reaches its zenith at the G2/M phase [20]. In sharp contrast, PLK1 protein expression remains unchanged during mouse oocyte maturation [21]. Inhibited PLK1 activity leads to multiple mitotic defects, including the formation of abnormal spindles, misaligned chromosomes, and improper chromosomal condensation [22]; while aberrant expression of PLK1 can induce diverse human tumor cell types [23, 24]. PLK1 becomes activated at meiotic resumption on MTOCs and later at kinetochore-microtubule (K-MT) attachments [25], and PLK1-induced BubR1 hyper-phosphorylation was found to be important for the establishment of stable K-MT interactions during chromosome congression [26]. Proteolysis of PLK1 is mediated by the anaphase-promoting complex/cyclosome (APC/C) ubiquitin ligase and requires the activating subunit Cdh1 [27]. An exquisite balance of PLK1 and its kinase activity is required for mouse oocyte chromosomal alignment, faithful mitotic progression, and euploidy [28]. PLK1 is also the key kinase involved in the regulation of MTOCs [29], and deregulation of PLK1 results in chromosomal instability and aneuploidy [30]. PLK1-null mutations are embryonically lethal, and the conditional deletion of *Plk1* either induces failure of growing oocytes to organize their α-tubulin or facilitates the development of abnormally small bipolar spindles [31].

We herein examined the subcellular distribution, expression, and function of PAK2 during mouse oocyte maturation. We demonstrated that *Pak2*-knockdown (KD) oocytes were arrested at the MI stage, and we elucidated a possible underlying molecular mechanism whereby PAK2 protein physically interacted with PLK1 protein, showing that *Pak2* depletion induced PLK1 protein degradation via APC/C^Cdh1^ pathway.

## Results

### Subcellular localization and expression of PAK2 during oocyte maturation

**Fig. 1:**
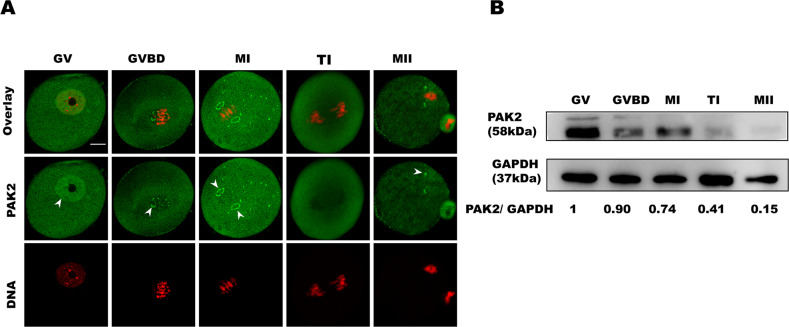
Subcellular localization and expression of PAK2 during oocyte maturation. **A** Confocal microscopy showing immunostaining for PAK2 (green) and DNA (red) in mouse oocytes at GV (germinal vesicle), GVBD (germinal vesicle breakdown), MI (metaphase I), TI (telophase I), and MII (metaphase II) stages. **B** Expression of PAK2 during meiotic maturation at GV, GVBD, MI, TI, and MII stages (the molecular mass of PAK2 is 58 kDa). Proteins from 200 oocytes were loaded for each sample (scale bar = 20 μm).

To investigate the function of PAK2 during meiotic maturation, we evaluated its subcellular localization and expression in mouse oocytes. Immunofluorescence and confocal imaging showed that PAK2 was predominantly distributed to the nucleus at the germinal vesicle (GV) stage (Fig. 1A, arrows), and that after GV breakdown (GVBD), PAK2 was localized to the cytoplasm and predominantly distributed around the chromosomes. However, commensurate with meiotic progression, PAK2 resided in the cytoplasm and significantly accumulated on the main MTOC region at the metaphase I and metaphase II stages (Fig. 1A, arrows). In addition, to confirm that the PAK2 antibody-staining pattern was specific, exogenous Myc-PAK2 was ectopically expressed in mouse oocytes (Fig. S1, A); and then GV, GVBD, and metaphase oocytes were labeled with anti-Myc antibody. We thus discerned that the Myc-PAK2 distribution pattern was similar to the localization of endogenous PAK2 protein (Fig. S1, B). PAK2 protein was expressed at the GV stage and gradually diminished during oocyte maturation. Collectively, the dynamic distribution and expression pattern imply that PAK2 plays a unique role in regulating oocyte meiotic maturation.

### *Pak2*-KD adversely affects meiotic progression in oocytes

**Fig. 2:**
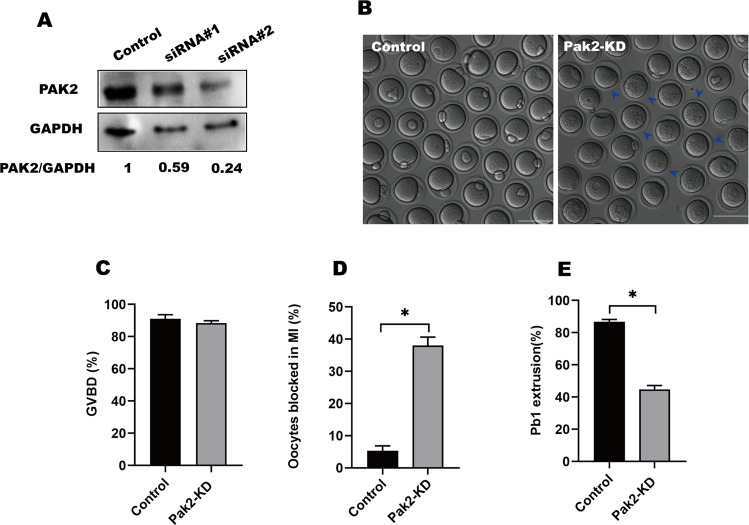
*Pak2*-knockdown (KD) adversely affects meiotic progression in oocytes. **A** KD of endogenous PAK2 protein after *Pak2*-siRNA injection was confirmed by western blot analysis. **B** Representative images of oocytes from control and *Pak2*-KD groups. Blue arrowheads indicate oocytes that were arrested at metaphase I and failed to extrude polar bodies (scale bar, 80 µm). **C** Quantitative analysis of GVBD rate between control (*n* = 120) and *Pak2*-KD (*n* = 108) oocytes (91.0 ± 2.16%, *n* = 120; 88.3 ± 1.25, *n* = 108, respectively). **D** Percentage of meiosis I-arrested oocytes after *Pak2*-siRNA injection (5.3 ± 1.25%, *n* = 120, control; 38.0 ± 2.16%, *n* = 108, *Pak2*-KD). **E** Quantitative analysis of Pb1 extrusion rate between control and *Pak2*-KD oocytes (86.7 ± 1.25%, *n* = 120; 44.7 ± 2.05%, *n* = 108, respectively). The graph shows the mean percentage ± SD of the results obtained from three independent experiments. *Significantly different (*p* < 0.05).

The specific localization and expression patterns of PAK2 in the meiotic process prompted us to investigate whether its KD affected the meiotic apparatus in oocytes. To address this question, specifically designed *Pak2*-siRNAs were microinjected into fully grown GV oocytes, with a negative siRNA injected as control. After injection, oocytes were arrested at the GV stage for 20 h in M16 medium containing 2.5 μM milrinone to allow the degradation of endogenous *Pak2* mRNA. Based on western immunoblotting analysis, we found that siRNA#2 induced the most substantial reduction in PAK2 protein in oocytes (Fig. 2A); similarly, siRNA#2-injected oocytes displayed a wide variation in PAK2 staining (Fig. S2). We, therefore, used siRNA#2 in subsequent KD experiments. As immunofluorescence and confocal imaging revealed that PAK2 was predominantly localized on the MTOC region (Fig. S2, A), we investigated whether *Pak2* depletion would affect the MTOC component. As MTOCs contain many of the pericentriolar material components—including BubR1, aurora A, PLK1, and pericentrin (PCNT) [29]—our quantitative real-time PCR (QPCR) results depicted *Bubr1*, *aurora A*, *Plk1* and *PCNT* mRNA levels have no significant change when Pak2 was depleted (Fig. S2, D–G).

After 3 h of culture, both control and *Pak2*-KD oocytes resumed meiosis (Fig. 2C). However, after 14 h of culture, only 44.5% of the *Pak2*-KD oocytes extruded Pb1, which was significantly less than the rate for controls (Fig. 2B, E, arrows). In addition, nuclear staining and quantitative analysis revealed that 38.0% of *Pak2*-KD oocytes were arrested at MI (Fig. 2D). Collectively, these results suggest that *Pak2*-KD disturbs meiotic progression during mouse oocyte maturation.

### PAK2 is essential for chromosome alignment in metaphase I oocytes

**Fig. 3:**
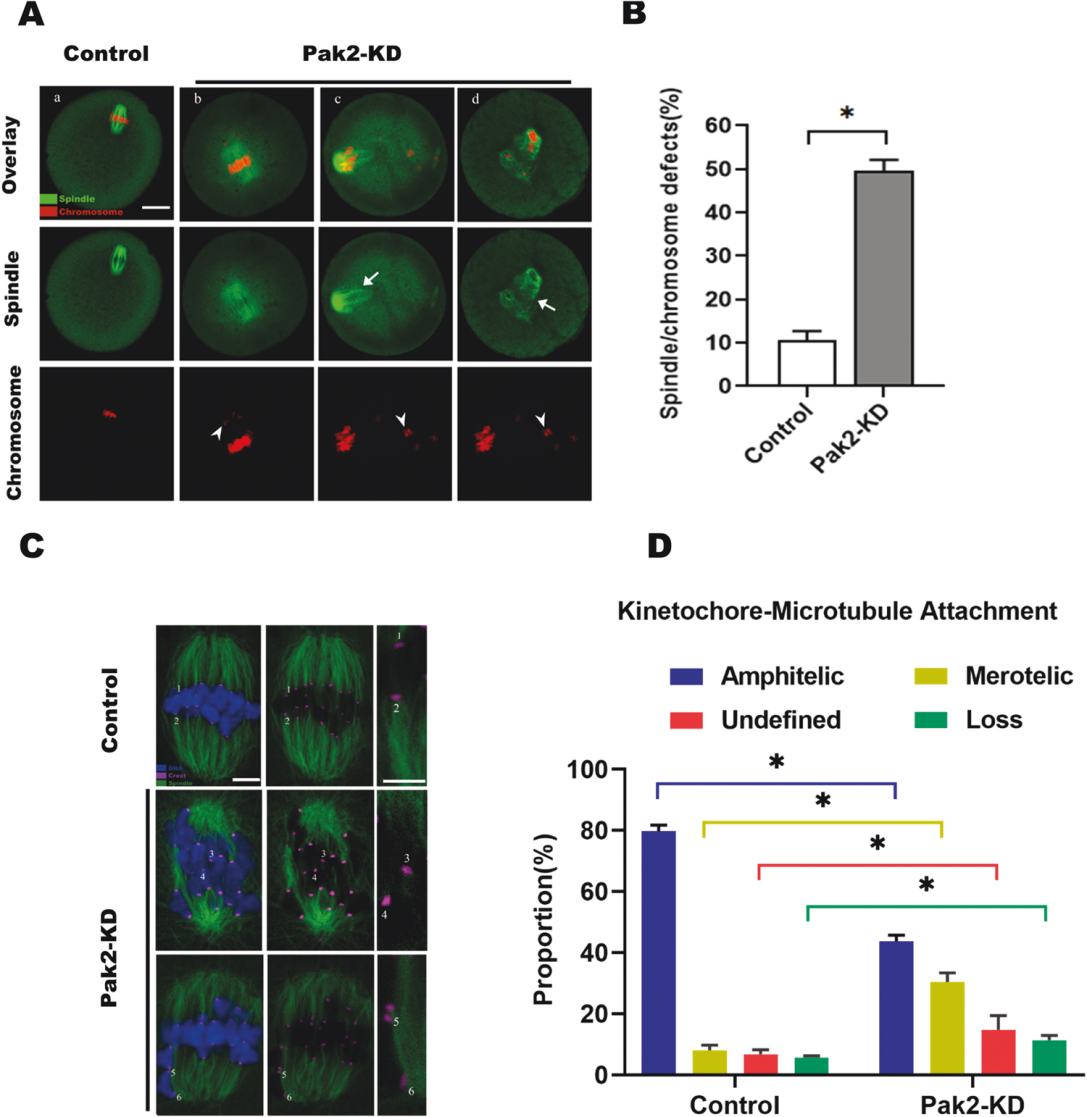
PAK2 is essential for meiotic apparatus organization. **A** Control and *Pak2*-KD oocytes were stained with α‐tubulin antibody to visualize the spindle (green) and counterstained with PI to observe chromosomes (red). (a) Control oocytes show the characteristic barrel-shaped spindle and well-aligned chromosomes. (b–d) Three examples illustrating the disorganized spindles (arrows) and misaligned chromosomes (arrowheads) that were frequently observed in *Pak2*-KD oocytes (scale bar, 20 μm). **B** Quantification of control and *Pak2*-KD oocytes with spindle/chromosome defects (10.7 ± 1.70%, *n* = 116; 49.7 ± 2.05%, *n* = 102, respectively). **C** Control and *Pak2*-KD metaphase oocytes were labeled with CREST antibody for kinetochores (purple), anti-tubulin antibody for microtubules (green), and Hoechst 33342 for chromosomes (blue) (representative confocal images are shown). Chromosomes labeled 1 and 2 represent examples of amphitelic attachment (79.7 ± 1.70%, *n* = 42, control; 43.7 ± 1.70%, *n* = 47, *Pak2*-KD), chromosomes 3 and 4 represent lost attachment (5.7 ± 0.47%, *n* = 42, control; 11.3 ± 1.25%, *n* = 47, *Pak2*-KD), chromosome 5 represents merotelic attachment (8.0 ± 1.41%, *n* = 42, control; 30.3 ± 2.49%, *n* = 47, *Pak2*-KD), and chromosome 6 represents undefined attachment (8.0 ± 1.41%, *n* = 42, control; 30.3 ± 2.49%, *n* = 47, *Pak2*-KD) (scale bar, 5 μm). **D** Quantitative analysis of K-MT attachments in control and *Pak2*-KD oocytes (kinetochores in regions where fibers were not easily visualized were not included in the analysis). Graph shows the mean percentage±SD of the results obtained from three independent experiments. *Significantly different (*p* < 0.05).

The specific localization of PAK2 during oocyte maturation prompted us to ask whether *Pak2* functions in the chromosome alignment. To gain insight into this issue, control and Pak2-KD oocytes were labeled with anti-tubulin antibody to visualize the spindle and co-stained with PI for chromosomes. Most control metaphase oocytes presented a typical barrel-shaped spindle and exhibited well-aligned chromosomes at the equatorial plate (Fig. 3Aa). In contradistinction, *Pak2*-KD oocytes frequently showed chromosomal misalignment (arrows) and spindle disorganization (arrowheads) (Fig. 3Abcd), and using quantitative analysis we noted that the proportion of *Pak2*-KD oocytes with spindle/chromosome defects was significantly higher than in control oocytes (Fig. 3B).

Precise chromosomal alignment at the equator and chromosome segregation depend upon the suitable attachment of kinetochores to microtubules that emanate from the opposite spindle pole [32]. Given the disorganization of chromosome/spindle in *Pak2*-KD oocytes, we speculated that reduced PAK2 affected K-MT attachments, and we visualized the kinetochores, MTs, and chromosomes of MI oocytes by staining with anti-CREST antibody, anti-tubulin antibody, and Hoechst 33342, respectively (Fig. 3C). In the majority of control oocytes, kinetochores were properly attached to MTs and presented amphitelic K-MT attachments (i.e., every kinetochore was attached to one pole; chromosomes are labeled 1 and 2 in Fig. 3C, D). In contrast, we determined an increased incidence of misattachments in *Pak2*-KD oocytes relative to control oocytes—including merotelic attachment (one kinetochore attached to both poles; chromosomes 3,4), undefined attachment (chromosome 5), as well as lost attachments (kinetochore attached to neither of the poles; chromosome 6). These erroneous K-MT attachments were likely the major factor contributing to the chromosomal alignment failure observed in *Pak2*-KD oocytes and thereby influenced the establishment of stable chromosomal biorientation. Together, these findings suggest that PAK2 fulfills a vital role in microtubular stability and chromosomal organization during mouse oocyte maturation.

### Reduced PAK2 activates the SAC and increases the incidence of aneuploidy

**Fig. 4:**
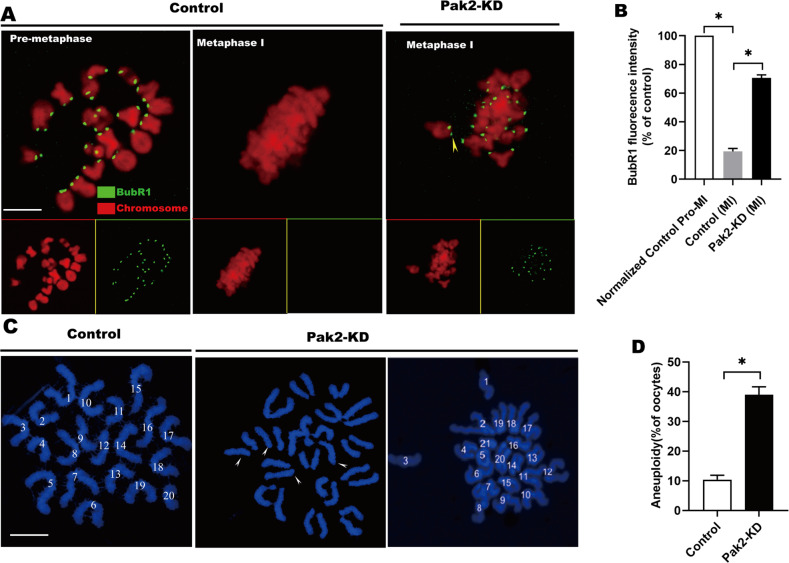
Reduced PAK2 activates the SAC and increases the incidence of aneuploidy. **A** Control and *Pak2*-KD oocytes were immunolabeled with anti-BubR1 antibody (green) and counterstained with PI to examine chromosomes (red). Representative confocal images of pre-MI and MI oocytes are shown. Arrowheads indicate scattered chromosomes in *Pak2*-KD oocytes (scale bar, 2.5 μm). **B** Quantification of BubR1 fluorescence intensity in control and *Pak2*-KD oocytes (19.3 ± 1.70%, *n* = 46, control; 70.7 ± 1.70%, *n* = 42, *Pak2*-KD). **C** Chromosome spreads of control and *Pak2*-KD MII oocytes (chromosomes were stained with Hoechst 33342 [blue]). Representative confocal images show euploid control oocytes and aneuploid *Pak2*-KD oocytes. Arrows indicate the premature separation of sister chromatids (scale bar, 2.5 μm). **D** Quantification of aneuploidy in control (*n* = 43) and *Pak2*-KD (*n* = 50) oocytes (10.3 ± 1.25%, *n* = 43, control; 39.0 ± 2.16%, *n* = 50, *Pak2*-KD). Graph shows the mean ± SD of the results obtained from three independent experiments. *Significantly different (*p* < 0.05).

The SAC is a ubiquitous surveillance system that monitors K-MT interactions and ensures accurate chromosome segregation [33], and steady K-MT attachment is indispensable to the SAC [33]. Once the K-MT attachments are disturbed, the anaphase-inhibitory signal is triggered by the SAC [34]. Taking into account the impaired K-MT attachments and MI arrest in *Pak2*-KD oocytes, we surmised that the SAC might be provoked when *Pak2* was depleted. To test this, control and *Pak2*-KD oocytes were immunolabeled for BubR1 (an integral component of the checkpoint complex) to evaluate SAC activity. In control oocytes, BubR1 was found on unattached kinetochores during pre-metaphase, and then completely disappeared once kinetochores became correctly attached to the microtubules at metaphase I (Fig. 4A). Intriguingly, the BubR1 signal on kinetochores was dramatically augmented in the *Pak2*-KD oocytes arrested at MI (Fig. 4A, B, arrowhead), implying that SAC was activated. These findings suggest that the SAC surveillance mechanism constitutes a major pathway that mediates the effects of *Pak2*-KD on meiotic progression in oocytes.

Dysregulated SAC is posited to be the major driving force of aneuploidy generation [35]. Due to the high frequency of spindle/chromosome defects in *Pak2*-KD oocytes, we then examined whether the incidence of aneuploidy was enhanced, and processed MII oocytes for chromosomal spreading (representative images of euploidy and aneuploidy are shown in Fig. 4C, and we also observed monosomy in *Pak2*-KD oocytes [Fig. 4C, arrows]). Quantitative analysis (Fig. 4D) revealed an approximately three- to four-fold increase in the incidence of aneuploid eggs compared with controls in the presence of *Pak2*-KD. These findings suggest that *Pak2*-KD disrupts spindle/chromosome organization and provokes SAC during meiosis, which in turn elevates the incidence of aneuploidy.

### PAK2 is associated with centrosomal protein PLK1

**Fig. 5:**
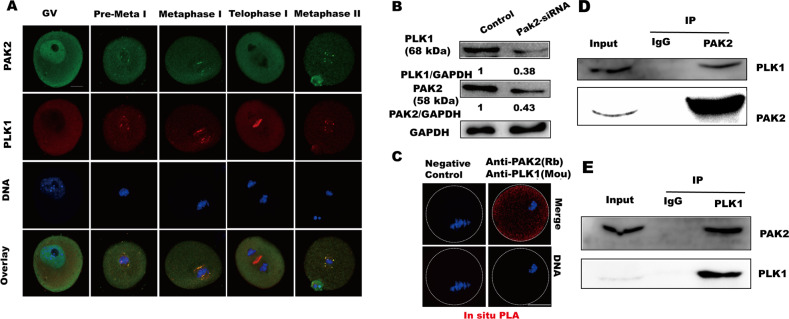
PAK2 is associated with centrosomal protein PLK1. **A** Confocal microscopy shows immunostaining for PAK2 (green), PLK1 (red), and DNA (blue) in mouse oocytes at the GV (germinal vesicle), GVBD (germinal vesicle breakdown), MI (metaphase I), TI (telophase I), and MII (metaphase II) stages (scale bar, 20 μm). **B** PLK1 protein levels in control and *Pak2*-KD oocytes. **C** Oocytes at the MI stage were fixed and incubated with rabbit anti-PAK2 and rabbit anti-PLK1 antibodies, followed by in situ PLA analysis; oocytes injected with PAK2-siRNA were used as negative controls (scale bar, 20 μm). **D** Co-IP was performed to determine the interaction between PAK2 and PLK1. Oocytes lysates was incubated with IgG and anti-PAK2 antibody, followed by incubation with protein G beads. The blots of IP eluates were probed with anti-PAK2 and anti-PLK1 antibodies, respectively. **E** Reciprocal Co-IP was performed with IgG and anti-PLK1 antibody. The blots of IP eluates were probed with anti-PLK1 and anti-PAK2 antibodies, respectively.

Immunofluorescence and confocal imaging showed that PAK2 co-localized with PLK1 and accumulated on the spindle poles at pre-MI, MI, and MII stages during mouse oocyte maturation (Fig. 5A). We next evaluated PLK1 protein expression levels in *Pak2*-depleted oocytes via western blot analysis and observed that PLK1 was markedly reduced after *Pak2* depletion (Fig. 5B). Notably, the meiotic phenotypes of *Pak2*-KD oocytes were highly similar to those of oocytes exposed to PLK1 inhibition [25], and we conjectured that reduced PLK1 contributed to the meiotic defects associated with *Pak2* depletion. To further clarify the relationship between PAK2 and PLK1, we performed an in situ proximity ligation assay (PLA) that allows visualization of the in vivo interactions between two proteins [29]. Whereas no signal was detected in the negative control, strong positive signals were observed throughout the cytoplasm, indicating a direct interaction between PAK2 and PLK1 in mouse oocytes (Fig. 5C). In order to more rigorously confirm the interaction between PAK2 and PLK1, we did co-immunoprecipitation (IP) using oocyte extracts followed by western blotting. The results are shown (Fig. 5D), immunoblots after co-immunoprecipitation (IP) with an antibody to PAK2 documented that PLK1 was present in the protein precipitate instead of IgG control group. In reciprocal experiments, PAK2 was observed in the protein precipitate after treatment with PLK1 antibody instead of IgG group, confirming that PAK2 interacts with PLK1 in oocytes (Fig. 5D, E). Collectively, our results suggest that reciprocal interactions between PAK2 and PLK1 are essential for spindle assembly and chromosome alignment during meiotic maturation.

### PAK2 protects PLK1 from APC/C^Cdh1^-mediated degradation

**Fig. 6:**
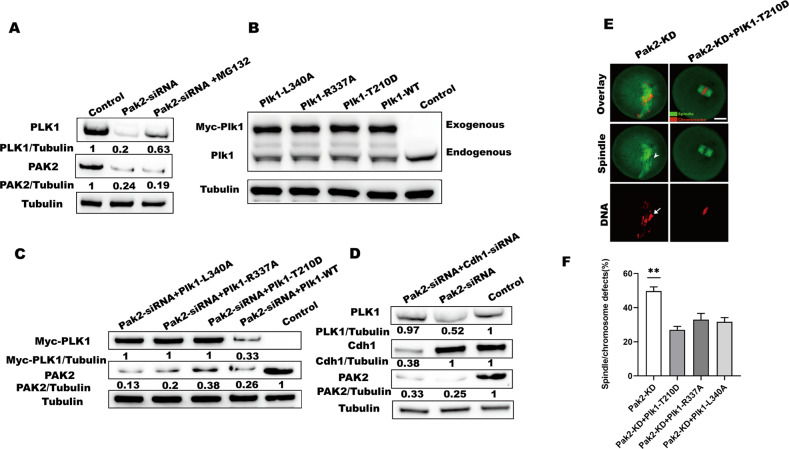
PAK2 protects PLK1 from APC/C^Cdh1^-mediated degradation. **A** PLK1 protein levels in control, *Pak2*-KD, and *Pak2*-KD + MG132 oocytes. The blots were probed with PAK2, PLK1 and tubulin antibodies. **B** Western blots show that the various mutant PLK1 proteins were expressed to a similar extent. The blots were probed with PLK1 and tubulin antibodies. **C** PLK1 protein levels in control, Pak2-KD + Plk1-L340A, Pak2-KD + Plk1-R337A, Pak2-KD + T210D and Pak2-KD + Plk1-WT oocytes. The blots were probed with Myc, PAK2 and tubulin antibodies. **D** PLK1 protein levels in control, *Pak2*-KD, and *Pak2*-KD + *Cdh1*-KD oocytes. The blots were probed with PAK2, Cdh1, PLK1 and tubulin antibodies. **E** *Pak2*-KD and *Pak2*-KD + *Plk1*-T210D oocytes were stained with α‐tubulin antibody to visualize the spindle (green) and counterstained with PI to observe chromosomes (red). Arrowheads indicate the misaligned chromosomes, and arrows indicate the defective spindles (scale bar, 20 μm). **F** Quantification of *Pak2*-KD, *Pak2*-KD + *Plk1*-T210D, *Pak2*-KD + *Plk1*-R337A and *Pak2*-KD + *Plk1*-L340A oocytes with spindle/chromosome defects (49.7 ± 2.05%, *n* = 116; 27.0 ± 1.63%, *n* = 111; 33.0 ± 2.94%, *n* = 98; 31.7 ± 2.05%, *n* = 104; respectively). ^*^p < 0.05 vs. controls. (scale bar, 20 μm).

PLK1 is also a known substrate of the E3 ubiquitin ligase APC/C^Cdh1^ during prophase and early pro-metaphase of oocytes [36], and in response to genotoxic stress in G2, PLK1 was degraded via ubiquitin ligase APC/C^Cdh1^ to permit sufficient time for effective DNA repair [27]. After *Pak2* depletion, mRNA levels of *Plk1* were not markedly changed (Fig. S2, F), but protein levels were reduced significantly (Fig. 5B); and we, therefore, hypothesized that *Pak2* depletion activated ubiquitin-proteasome and mediated PLK1 degradation. To support our hypothesis, we inhibited the activity of ubiquitin proteasome in *Pak2*-depleted oocytes by MG132 (Sigma, St. Louis, USA; Cat# M8619, 5 μM), a proteasomal inhibitor; and we find that Plk1 protein in MG132 treated Pak2-depleted oocytes was partially recovered (Fig. 6A). In addition, T210D, R337A, and L340A *Plk1* mutants were reported to be stable during mitosis and not degraded by APC/C^Cdh1^ [27, 37, 38]. To ascertain whether the degradation of PLK1 was mediated by APC/C^Cdh1^, we constructed site-specific mutants (T-to-D, R-to-A, and L-to-A) that targeted T210, R337, and L340, respectively. Fully grown GV oocytes were microinjected with the mRNA for each specific *Plk1* mutant, *Plk1*-WT, or control PBS (immunoblotting verified that exogenous PLK1 protein was efficiently produced in the mouse oocytes); and the various mutant PLK1 proteins were expressed to similar extents (Fig. 6B). Subsequently, *Pak2*-siRNA were microinjected into control, *Plk1*-WT and all *Plk1* mutant-overexpressing oocytes to observe the protein levels of exogenous Myc-PLK1. Consistent with our hypothesis, exogenous PLK1 protein was markedly reduced in the *Pak2*-siRNA + *Plk1*-WT group but remained unaltered in the *Pak2*-siRNA + *Plk1* mutant groups (Fig. 6C). To further discern whether the degradation of PLK1 was mediated by APC/C^Cdh1^, GV oocytes were microinjected with Pak2-siRNA or with PBS as a control, and *Pak2*-KD GV oocytes were microinjected with *Cdh1* siRNA; we thereby proved that PLK1 protein was partially recovered in the *Pak2*-siRNA + *Cdh1*-siRNA-injected oocytes (Fig. 6D). This suggested that reduced PLK1 in response to *Pak2*-KD was due to increased APC/C^Cdh1^ activity. In addition, ectopic expression of *Plk1*-T210D alleviated the spindle/chromosome defects (Fig. 6E, F) in *Pak2*-KD oocytes. Our data showed that *Pak2-*KD-induced meiotic defects are partially due to APC/C^Cdh1^-mediated PLK1 protein degradation.

## Discussion

Emerging evidence shows that *Pak2* participates in diverse biological processes [12], cytoskeletal dynamics [39], and DNA lesions in particular [40]. In the present study, we observed a dynamic localization of PAK2 during meiotic progression of mouse oocytes. We demonstrated that PAK2 predominantly accumulated on the spindle poles in concert with meiotic resumption (Fig. 1A), and that PAK2 protein expression levels that were noted at the GV stage gradually diminished during oocyte maturation (Fig. 1B). These unique localization and expression dynamics of PAK2 in oocytes imply that PAK2 participates in distinct or additional functions in meiosis.

In support of this speculation, we further confirmed that *Pak2*-KD in mouse oocytes disrupted spindle assembly and chromosomal alignment, and impaired K-MT interactions (Fig. 3). Our previous investigation also revealed that silencing of *Pak2* in early embryos of mice precipitated defects in spindle assembly and in chromosomal alignment [41]. As faithful chromosome separation is ensured by the bi-oriented interaction of chromosomes to the spindle through the end-on attachment of microtubules to kinetochores [42], it is conceivable that the high percentage of spindle/chromosome abnormalities in *Pak2*-KD oocytes is a consequence of K-MT misattachments. If these attachment errors are not corrected prior to anaphase in normal oocyte maturation, they may cause chromosome-separation defects [43]; and consistent with this concept, the frequency of aneuploidy was significantly increased in *Pak2*-depleted oocytes relative to controls (Fig. 4). We thereby proposed that compromised K-MT stability in *Pak2*-KD oocytes might, at least in part, contribute to the meiotic defects in and the generation of the aneuploid eggs observed in our experiments.

Investigators recently noted that inactivation of *Pak2* caused oxidative stress and DNA lesions [41, 44]. Upon DNA damage, proliferating cells initiate a regulatory signaling network to either delay the cell cycle or enable DNA repair [45], and it was shown that the ubiquitin ligase APC/C^Cdh1^ was activated by Cdc14B with DNA lesioning by subsequent degradation of PLK1 to allow sufficient time for effective DNA repair [27]. APC/C^Cdh1^-mediated proteolysis of PLK1 was reported in some somatic cells and oocytes after DNA damage, and led to withdrawal from the cell cycle [27, 46]. Our recent data also verified that *Pak2*-KD in early mouse embryos led to distinct embryonic DNA damage [41]. *Pak2*-KD oocytes exhibited a phenotype highly similar to that with *Plk1* inhibition [25], and *Plk1* was depicted to be a direct target of the G2 DNA-damage checkpoint [47]. Given the changes in *Plk1* mRNA and protein levels in *Pak2*-KD and control oocytes and the observation that most *Pak2*-KD oocytes were arrested at MI, we speculated that *Pak2* depletion induced DNA lesions and then activated APC/C^Cdh1^; this would result in the degradation of PLK1, ultimately inducing oocyte developmental arrest and aberrations in meiotic apparatus assembly. Our immunofluorescence results revealed that PAK2 co-localized with PLK1 at pre-MI, MI, and MII stages during mouse oocyte maturation (Fig. 5A), and that *Pak2* depletion induced a marked attenuation in PLK1 protein (Fig. 5B). In situ PLA and co-IP data confirmed a direct interaction between PAK2 and PLK1 (Fig. 5C, D, E), verifying that PAK2 was required to preserve PLK1 protein levels in mouse oocytes. The proteasomal inhibitor MG132 (Fig. 6A) and *Plk1* site-specific mutants (Fig. 6C) restored PLK1 protein levels in *Pak2*-depletion oocytes, and PLK1 protein levels remained steady in *Pak2*-siRNA + *Cdh1*-siRNA oocytes (Fig. 6D). PLK1 degradation was eliminated when siRNA reduced the level of Cdh1, suggesting that Cdh1 promoted PLK1 degradation. This further confirmed that MI arrest and meiotic apparatus assembly defects in *Pak2*-depleted oocytes were due to the degradation of PLK1 via APC/C^Cdh1^. However, our work did not preclude other pathways from being involved in this process.

**Fig. 7:**
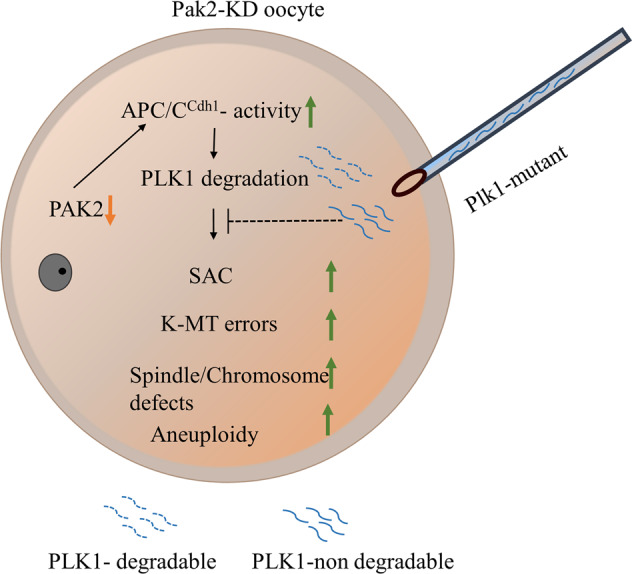
Proposed model of PAK2 in meiotic progression and meiotic apparatus assembly in mice oocytes. When PAK2 level reduced in oocytes, incerased APC/C^Cdh1^ activity promotes PLK1 degradation and thus cause oocyte meiotic abnormality. Microinjection of nondegradable Plk1-mutant in vitro can partially rescued these errors.

Researchers demonstrated that normal cell division was exceedingly dependent upon tightly controlled PLK1 expression levels and kinase activity [43, 48, 49], and a precise regulation of PLK1 protein and its kinase activity was required for chromosomal alignment and precise mitotic progression [50]. PAK activity is required in HeLa cells for their entry into mitosis and proper spindle formation, and this is at least partially due to PAK regulation of PLK1 [17]. In vitro kinase assays demonstrated that the expression of PAK was positively correlated with the activity of PLK1 [17], and down-regulation of PLK1 or pharmacological inhibition of its kinase activity led to mitotic defects and activation of the SAC and apoptotic death [28, 51, 52]. Our results likewise showed that the *Pak2*-KD oocytes suffered severe spindle-assembly defects (Fig. 3Ab), chromosomal aberrations (Fig. 3Acd), and SAC-signal activation (Fig. 4A). PLK1 is needed to recruit centrosomal proteins to aMTOCs so as to promote normal spindle formation and is required for stable K-MT attachment, and the loss of PLK1 kinase activity leads to metaphase I arrest with misaligned chromosomes activating the SAC [22, 53] (Fig. 7).

In summary, our results revealed that PAK2 is a cytoskeletal regulator required for meiotic maturation in mouse oocytes. We observed that defects in the chromosome alignment and MI arrest of *Pak2*-depleted oocytes were partially due to reduced PLK1, and that PAK2 protected PLK1 from APC/C^Cdh1^-mediated degradation (Fig. 7).

## Materials and methods

All chemicals and culture media were purchased from Sigma (St. Louis, MO, USA) unless stated otherwise.

### Mice

The mice used in this study were purchased from Beijing Weitong Lihua Co., Ltd. and raised in the SPF Animal Center of the School of Life Sciences, Sun Yat-sen University. All experimental animal protocols were performed in accordance with relevant ethical guidelines and regulations, and approved by the Third Affiliated Hospital of Guangzhou Medical University.

### Antibodies

Rabbit polyclonal anti-PAK2 (Cat# ab76293), anti-PLK1 (Cat# ab17056), and anti-BubR1 antibodies (Cat# ab254326) were obtained from Abcam (Cambridge, MA, USA); anti-Cdh1 antibody (Cat# sc-56312) from Santa Cruz Biotechnology (Santa Cruz, CA, USA); mouse monoclonal α-tubulin-FITC antibody (Cat# F2168) from Sigma (St. Louis, MO, USA); anti-C-myc-antibody (Cat# 2278 S), HRP-conjugated rabbit (Cat #7074 S) and mouse (Cat# 7076 S) secondary antibodies were from Cell Signaling Technology (Danvers, MA, USA); GAPDH monoclonal antibody (Cat# 60004-1 g); Polyclonal Antibody Tubulin (11224-1-AP); Rabbit IgG (30000-0-AP) and Mouse IgG (B900620) were purchased from Proteintech (Wuhan, China); human anticentromere CREST antibody (Cat:#15-234) from Antibodies Incorporated (Davis, CA); Fluor 555 (Cat# A-11001) and 488 (Cat# A-21429) conjugated anti-mouse and anti-rabbit secondary antibodies from Invitrogen (Invitrogen, USA); and Cy5‐conjugated donkey anti‐human IgG (Cat #709-605-149) was purchased from Jackson Immuno Research Laboratory (West Grove, PA), Mouse anti-Rabbit IgG (Light Chain specific),HRP (BE0107-100) and Goat anti-Mouse IgG (Light Chain specific),HRP (BE0105-100) was purchased from Easybio (Beijing, China).

### Oocyte collection and culture

To obtain fully grown GV oocytes, mice were superovulated with 5 IU of equine chorionic gonadotropin (eCG; Teikoku Zoki, Tokyo, Japan) by intraperitoneal injection; 48 h later, GV oocytes were harvested from the ovaries at 6–8 weeks of age mice, and placed in M2 (Sigma, St. Louis, USA; Cat# M7167) medium supplemented with 2.5 μM milrinone to maintain oocyte arrest at the GV stage. Oocytes were subsequently cultured in mini-drops of M16 (Sigma, St. Louis, USA; Cat# MR-016) medium covered with mineral oil (Sigma, St. Louis, USA; Cat# M8410) at 37 °C in an atmosphere of 5% CO_2_ in humidified, compressed air.

### Plasmid construction and mRNA synthesis

Total RNA was extracted from 50 denuded oocytes at the stages indicated above using the Arcturus PicoPure RNA Isolation Kit (Applied Biosystems, CA, United States), and cDNA was generated with the Quantitect Purification Kit (Qiagen, Düsseldorf, Germany) (the primers we used to amplify the coding DNA sequence [CDS] of *Plk1* and mutants are listed in Table S1 [Supporting Information]). PCR products were purified, digested with Fse I and Asc I (New England Biolabs, Beverly, MA, USA), and then inserted into the pCS^2+^ vector with Myc-tags. For the synthesis of cRNA Myc-*Pak2* mRNA, plasmid constructs were linearized using Not1 enzyme (NEB) and purified. Capped cRNAs were constructed using in vitro transcription with SP6 mMESSAGE mMACHINE (Ambion, CA, USA) according to the manufacturer’s instructions. Synthesized RNA was ultimately aliquoted and stored at −80 °C.

### Sit‐directed mutagenesis

We employed wild-type *Plk1* cloned onto the expression plasmid pcs^2+^ to individually generate the T210D, R337A, and L340A mutants. To introduce the mutation, we executed high-fidelity inverse PCR using divergent primers with one per pair being mutagenic (Table S1). Template DNA was eliminated by DpnI digestion, and the resultant products were then circularized by performing a blunt-end ligation and transformed into *E. coli* TOP10. The mutant sequences were verified by DNA sequencing.

### KD and overexpression analysis

We microinjected siRNA or mRNA with an Eppendorf microinjector to knock down or overexpress specific proteins in mouse oocytes, respectively. For the RNAi experiment, siRNAs were diluted to 1 mM with RNase‐free ddH2O and stored in a − 80 °C refrigerator. The siRNAs were then diluted to 20 μM, and fully grown GV-intact oocytes were microinjected with 7–10 pL of non-targeting (control) or targeting siRNA in M16 medium in the presence of 2.5 μM milrinone. For overexpression experiments, 10 pl of mRNA solution (10 ng/l) was injected into the cytoplasm of GV oocytes; and the same amount of RNase‐free PBS was injected into controls. After cRNA (or siRNA) injection, oocytes were arrested at the GV stage in M16 medium containing 2.5 μM milrinone for 4 or 20 h to allow time for siRNA-mediated KD or to permit overexpression, respectively. The oocytes were then transferred to milrinone-free medium for further experimentation (the siRNA [cRNA] pairs that we used are listed in Supporting Information Table S2).

### Quantitative real-time PCR

Total RNA was extracted from 50 oocytes using the RNeasy® Micro Kit (Cat: #157030297, Invitrogen, USA), and cDNA synthesis was accomplished using a QuanNova Reverse Transcription Kit (Cat: #205311, Qiagen, Germany). QPCR was conducted using a Power SYBR Green PCR Master Mix (Applied Biosystems, Life Technologies) with an ABI 7500 Real-Time PCR system (Applied Biosystems). Data were normalized against Tubulin and quantification of fold-change in expression was determined using the comparative CT method as we reported previously [54] (the relevant primers are listed in Table S3).

### Immunoprecipitation

1500–2000 MI oocytes were put into RIPA Lysis Buffer contained phosphatase inhibitor cocktail (100×) (Kangwei Biotechnology, China), and were completely cleaved on ice block. We collected supernatant after centrifugation (12,000 rpm, 20 min) and we took out 50 μL of each supernatant as input sample at 4 °C. Another 500 μL was incubated with primary antibody (PAK2 or PLK1 antibody. PAK2, 1:200; PLK1, 1:1000) overnight at 4 °C. 50 μL of conjugated beads (washed five times in PBS) were added to the 500 μL supernatant/antibody mixture and incubated at 4 °C for overnight, after three times wash by immune complexes the samples were then released from the beads by mixing in 2× SDS loading buffer for 10 min at 100 °C. Supernate was mixed with Laemmli sample buffer to be used.

### Western blotting analysis

For total protein extraction, 200 oocytes from each group were heated at 100 °C for 5 min in protein lysis buffer (95% Laemmli sample buffer and 5% β-mercaptoethanol) and stored at −20 °C until used. Protein lysates (200 oocytes for each sample per lane) were separated on 12% SDS-PAGE gels and transferred to PVDF membranes. The membranes were blocked in 5% nonfat dry milk with PBS-Tween 20 (0.1%, vol/vol) for 1 h at room temperature (RT), and incubated with the primary antibodies overnight at 4 °C (PAK2 antibody, 1:1000; Myc antibody, 1:1000; PLK1 antibody, 1:2000; Tubulin antibody, 1:1000; GAPDH antibody, 1:1000). After three washes with PBST-Tween 20, membranes were further incubated with corresponding HRP-conjugated secondary antibodies for 1 h at RT. The protein bands were ultimately visualized with an ECL Plus Western Blotting Detection System (GE Healthcare, Piscataway, NJ, USA).

### Immunofluorescence

For immunofluorescence staining, oocytes were fixed in 4% paraformaldehyde (Sigma, St. Louis, USA; Cat# 158127-100 G) for 30 minutes, followed by permeabilization with 0.5% Triton X‐100 (Sigma, St. Louis, USA; Cat# T8787-100ML) for 20 min and blocking with BSA in PBS (1%, wt/vol) for 60 min. Oocytes were incubated at 4 °C overnight with primary antibodies in blocking solution (PAK2, 1:200; PLK1, 1:50; CREST, 1:300; and BubR1, 1:200). After washing three times with 1X wash buffer for 5 min each time, we allowed for the secondary antibody reaction with Alexa546 anti-rabbit (Invitrogen, A11071, × 1,000) or Dylite650 anti-mouse antibodies (Abcam, ab96784, × 1,000) in PBS at RT for 1 h. Chromosomes were counterstained with propidium iodide (PI, red) or Hoechst 33342 (blue) for 10 min. Finally, oocytes were mounted on anti-fade medium (Vectashield, Vector Laboratories, CA, USA) and then examined under a laser-scanning confocal microscope (LSM 710; Carl Zeiss, Germany). All immunofluorescence experiments were duplicated and repeated independently at least three times. ImageJ software (National Institutes of Health, USA) was used to quantify the intensity of fluorescence.

### Chromosome spreads

Chromosome spreads were performed as described previously [32]. Briefly, the zonae pellucidae of oocytes were removed with Tyrode’s acid solution (Sigma, St. Louis, USA; Cat# T1788-100ML) and transferred to M2 medium for recovery. Oocytes were then fixed in 1% paraformaldehyde containing 0.15% Triton X-100. After drying at RT, slides containing oocytes were blocked with 1% BSA in PBS for 1 h, nuclear DNA was stained using Hoechst 33342, and the samples were visualized using an inverted confocal microscope (LSM 710; Carl Zeiss, Germany) with a ×60 objective.

### Proximity ligation assay

PLA was performed using the in situ Red Starter Kit Mouse/Rabbit (Sigma, St. Louis, USA; Cat# DUO92101) to detect PAK2-PLK1 interactions using fluorescence microscopy according to the manufacturer’s protocol. Mouse anti-PLK1 and rabbit anti-PAK2 antibodies were conjugated with PLA PLUS and PLA MINUS probes, respectively, and oocytes injected with *Pak2*-siRNA were used as negative controls. The PLA signals were ultimately visualized using a confocal laser-scanning microscope (LSM 710; Carl Zeiss, Germany). One individual dot represented the close proximity of two interacting proteins within an oocyte, and the number of fluorescent foci per single oocyte was quantified using ImageJ software.

### Statistical analyses

We executed statistical analyses using Prism software, and graphical data are presented as means ± SD, unless otherwise indicated. Unpaired, two-tailed Student’s *t*-tests were used to assess differences between two groups; comparisons among more than two groups were analyzed with a one-way ANOVA with Tukey’s multiple comparisons using Prism. Each experiment was repeated at least three times, and *p* values≤0.05 were considered to be statistically significant.

## Supplementary information

Supplemental Figures and Tables

Supplemental Materials Uncropped WB

aj-checklist

## Data Availability

All data needed to evaluate the conclusions in the paper are present in the paper and/or the Supplementary Materials.
