# Supplementary material for: PAK2 is essential for chromosome alignment in metaphase I oocytes
Source: Cell Death Dis. 2023 Feb 22;14(2):150. doi: 10.1038/s41419-023-05585-7 (PMC9947007; doi:10.1038/s41419-023-05585-7)
Supplement: Supplementary file 1 — Supplemental Figures and Tables [file 41419_2023_5585_MOESM1_ESM.docx]

**Supplemental Figures and Tables for**

**PAK2 is essential for chromosome alignment in metaphase I oocytes**

Juan Zeng^1,2,3#^, Shiwei Wang^4#^, Min Gao^5#^, Dian Lu^1,2^, Shuang Song^4^, Diyu Chen^1,2^, Weimin Fan^1,2^, Zhiliang Xu ^1,2^, Zhiguo Zhang^3🖂^ , Xiaofang Sun^1,2🖂^

*Corresponding author: zhangzhiguo@ahmu.edu.cn

xiaofangsun@gzhmu.edu.cn

**This file includes:**

Figs. S1 to S2

Tables. S1 to S3

**
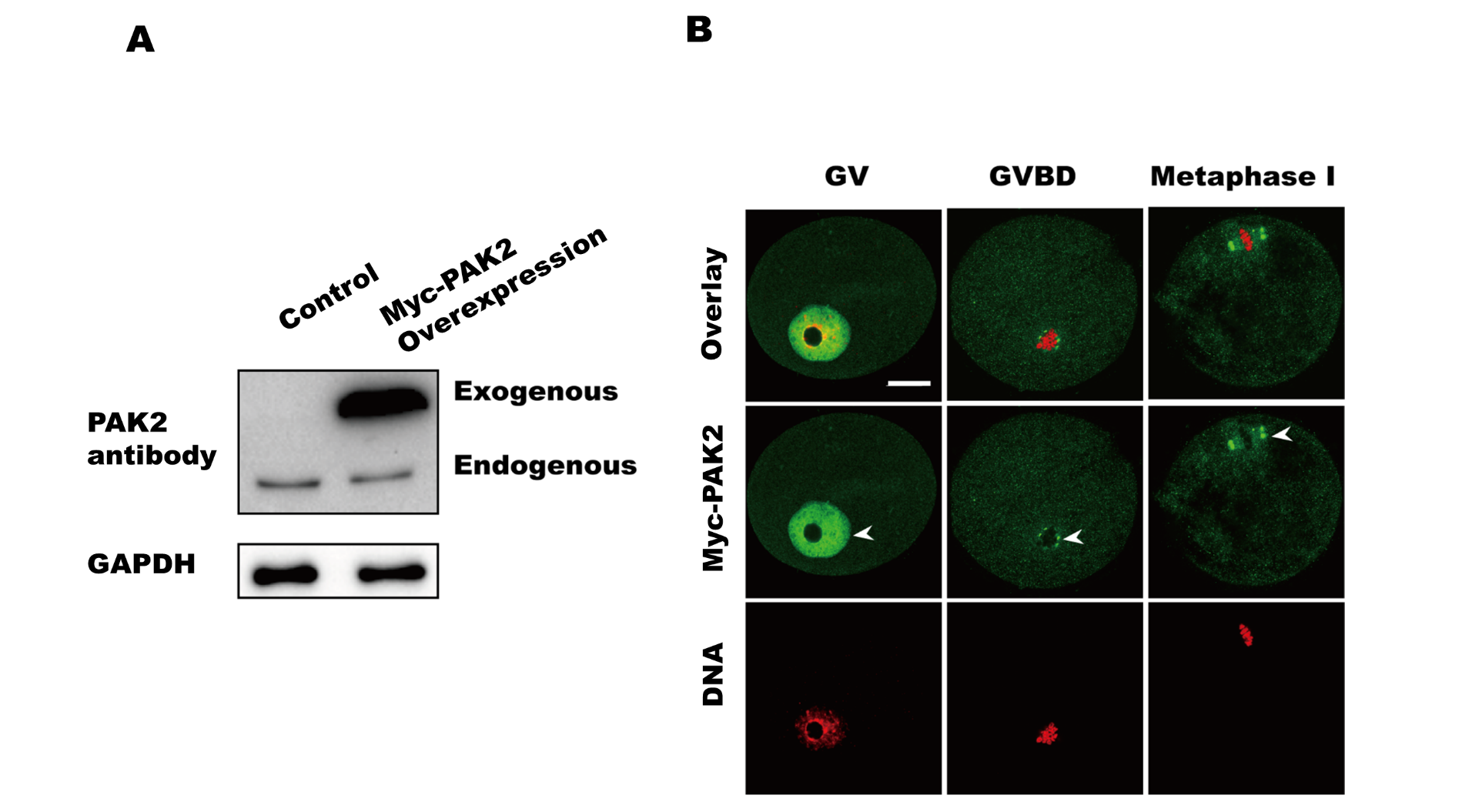
**

Fig. S1. Localization of Myc-PAK2 in mouse oocytes. (A) Representative western blot results show efficient overexpression of exogenous Pak2 protein. (B) Representative confocal sections show Myc-PAK2 cRNA-injected oocytes stained with anti-Myc antibody (green) and chromosomes counterstained with propidium iodide (red) (scale bar, 20 µm).

**
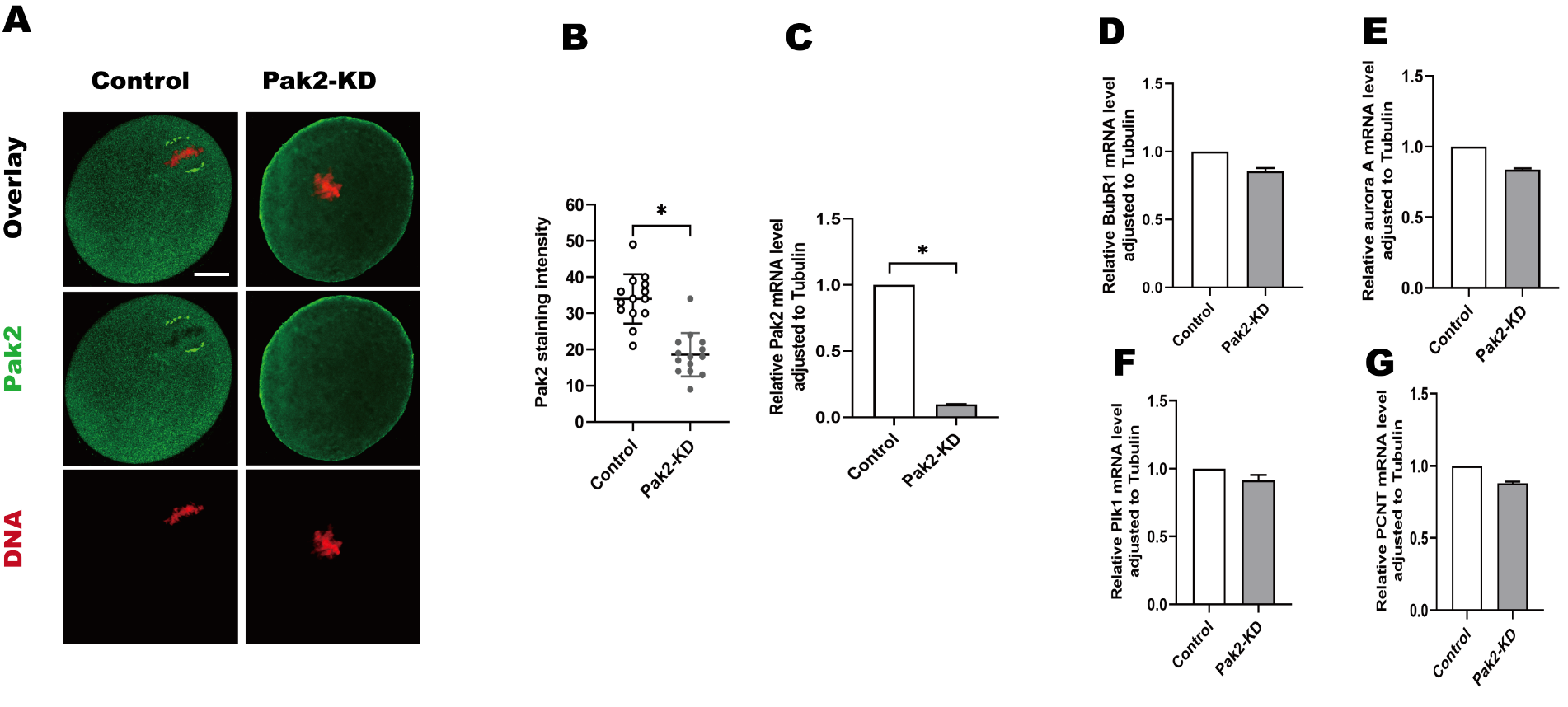
**

Fig. S2. Effects of siRNA KD on PAK2 staining in oocytes. (A) Representative confocal sections of control and *Pak2*-siRNA-injected oocytes stained with anti-PAK2 antibody (green) and chromosomes counterstained with propidium iodide (red) (scale bar, 20 µm). (B) Quantification of PAK2 immunofluorescence is shown in A (34.0 ± 6.56%, n = 15, control; 18.6 ± 5.78%, n = 15, *Pak2*-KD). (C-G) *Pak2, BubR1, aurora A, Plk1* and PCNT mRNA expression levels in *Pak2*-KD and control oocytes (n = 50 in each group). Experiments were repeated three times, and results are means ± SD. *p < 0.05.

Supplementary Table 1. Primer sequences used for cDNA amplification and site-directed mutagenesis.

| Gene | Primer sequence |
| --- | --- |
| Pak2-WT-F | 5’- CCGGAATTCGCTGGCTGTTTCATAATTC -3’ |
| Pak2-WT-R | 5’- AGGCGCGCCCTCGGTGACGATGTTAG-3’ |
| Plk1-WT-F | 5’-CCGGAATTCGACTCAGCCAGGTTCGTG -3’ |
| Plk1-WT-R | 5’-TGCTCTAGA CTAGCAGAGTGAAGGGGC -3’ |
| Plk1-T210D-F | 5’-AAGGGGAACGAAAGAAGGACTTGTGTGGCACTCC-3’ |
| Plk1-T210D-R | 5’-GGAGTGCCACACACAAGTCCTTCTTTCGTTCCCCTT-3’ |
| Plk1- R337A-F | 5’-GCCTGGACCCCAGCAGCGCGAAACCTCTCAAAGT-3’ |
| Plk1-R337A-R | 5’-GCCTGGACCCCAGCAGCGCGAAACCTCTCAAAGT-3’ |
| Plk1- L340A-F | 5’-CCAGCAGCAGGAAACCTGCCAAAGTCCTCAATAA-3’ |
| Plk1- L340A-R | 5’-TTATTGAGGACTTTGGCAGGTTTCCTGCTGCTGG-3’ |

Supplementary Table 2. Nucleotide sequences of *Pak2* and *Cdh1* siRNAs, and of the control siRNA.

| Gene | Sequence |
| --- | --- |
| Pak2-siRNA#1-F | 5’-GGUCCCAAAGUUGACAUAUTT-3 |
| Pak2-siRNA#1-R | 5’-AUAUGUCAACUUUGGGACCTT-3’ |
| Pak2-siRNA#2-F | 5’- CUUUAACUCUUUCAUCACC-3’ |
| Pak2-siRNA#2-R | 5’- GGUGAUGAAAGAGUUAAAG-3’ |
| Cdh1-siRNA-F | 5’-GCUACUCACAGAACCAGAUTT-3’ |
| Cdh1-siRNA-R | 5’-AUCUGGUUCUGUGAGUAGCTT-3’ |
| Control-siRNA-F | 5’-UUCUCCGAACGUGUCACGUTT-3’ |
| Control-siRNA-R | 5’-ACGUGACACGUUCGGAGAATT-3’ |

Supplementary Table 3. Primer sequences used for qPCR.

| Gene | Prime sequence |
| --- | --- |
| Pak2-qPCR-F | 5’-GCTGTAGTGACAGAGGAAGATGA-3 |
| Pak2-qPCR-R | 5’-TCACCAACTGGAGCAGGAATGG-3’ |
| BubR1-qPCR-F | 5’-GTCCACAGGTTCTCAATGCCCA-3’ |
| BubR1-qPCR-R | 5’-TGATGGCGTCTTCACTCAGAGG-3’ |
| Plk1-qPCR-F | 5’-CCATCTTCTGGGTCAGCAAGTG-3’ |
| Plk1-qPCR-R | 5’-CCGTCATTGTAGAGAATCAGGCG-3’ |
| aurora-A-qPCR-F | 5’-TCATCCTGGCTCTGAAGGTGCT-3’ |
| aurora-A-qPCR-R | 5’-CCATACAGCCTGAGGATGTTGG-3’ |
| PCNT-qPCR-F | 5’-GAGGAGAAGTCGGTCTTGTGGA-3’ |
| PCNT-qPCR-R | 5’-GCGGTCCTTTTCAGACTGCTTC-3’ |
| Tubulin-qPCR-F | 5’- GGCAGTGTTCGTAGACCTGGAA -3’ |
| Tubulin-qPCR-R | 5’- CTCCTTGCCAATGGTGTAGTGG -3’ |
