# Supplementary material for: PAK2 is essential for chromosome alignment in metaphase I oocytes
Source: Cell Death Dis. 2023 Feb 22;14(2):150. doi: 10.1038/s41419-023-05585-7 (PMC9947007; doi:10.1038/s41419-023-05585-7)
Supplement: Supplementary file 2 — Supplemental Materials Uncropped WB [file 41419_2023_5585_MOESM2_ESM.docx]

**Supplemental Materials Uncropped WB for**

**PAK2 is essential for chromosome alignment in metaphase I oocytes**

Juan Zeng^1,2,3#^, Shiwei Wang^4#^, Min Gao^5#^, Dian Lu^1,2^, Shuang Song^4^, Diyu Chen^1,2^, Weimin Fan^1,2^, Zhiliang Xu ^1,2^, Zhiguo Zhang^3🖂^ , Xiaofang Sun^1,2🖂^

*Corresponding author: zhangzhiguo@ahmu.edu.cn

xiaofangsun@gzhmu.edu.cn

**Original Western blot image**

**
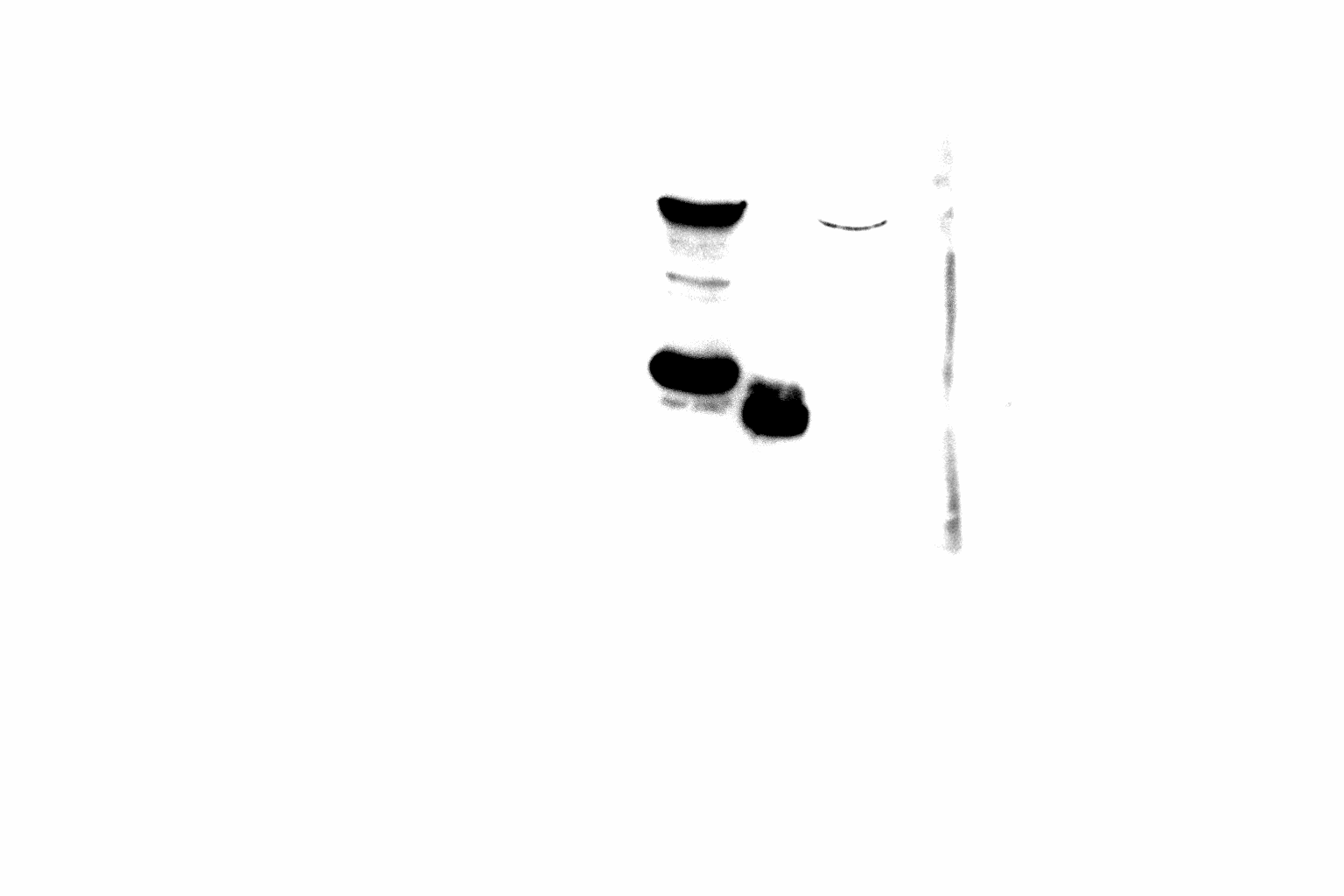
**





PAK2(58kDa)

GAPDH (37kDa)

Fig. 1. Subcellular localization and expression of PAK2 during oocyte maturation. (B) Expression of PAK2 during meiotic maturation at GV, pre-MI, MI, TI, and MII stages (the molecular mass of PAK2 is 58 kDa).


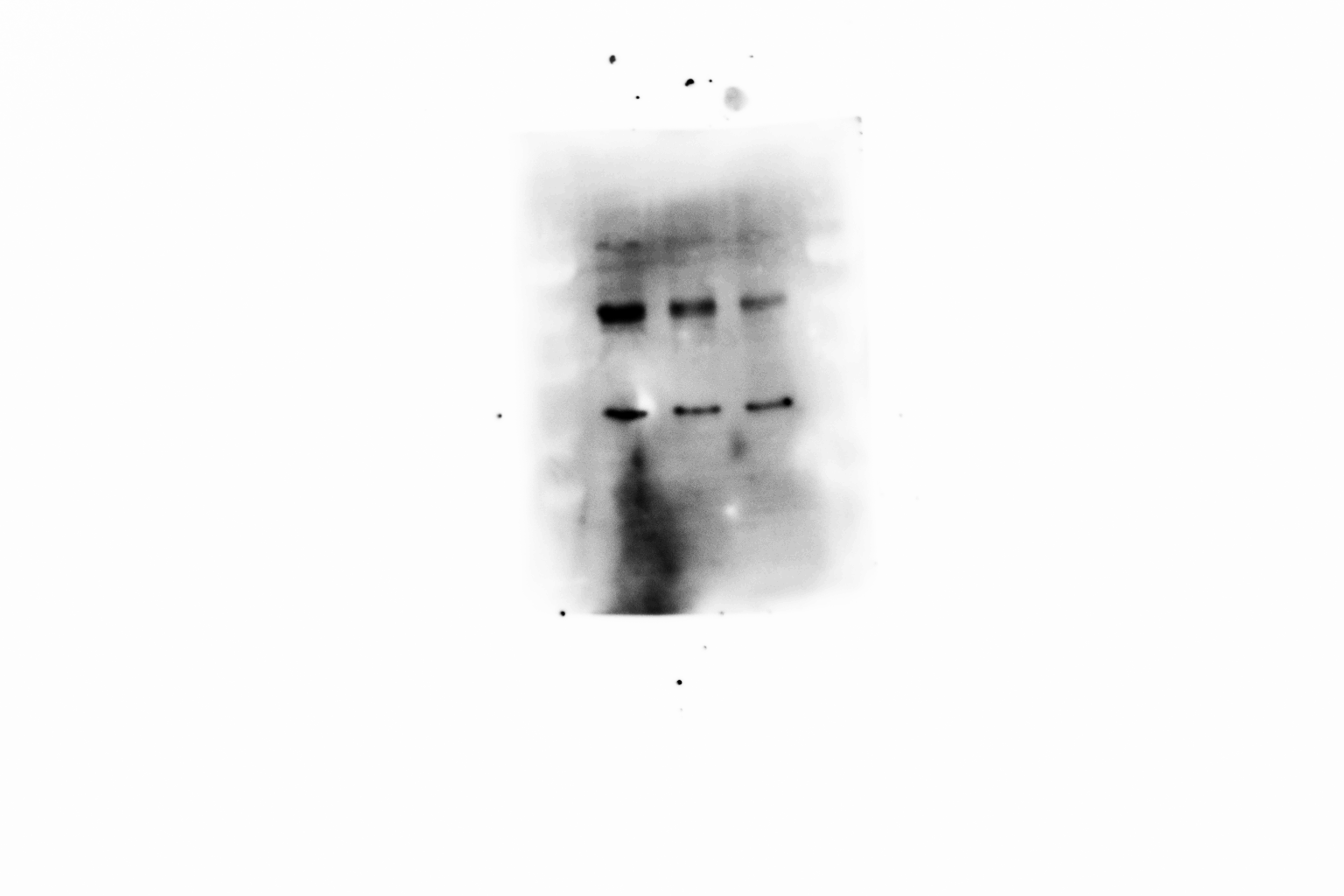


PAK2(58kDa)

GAPDH (37kDa)

Fig. 2. *Pak2*-knockdown (KD) adversely affects meiotic progression in oocytes. (A) KD of endogenous PAK2 protein after *Pak2*-siRNA injection was confirmed by western blot analysis.


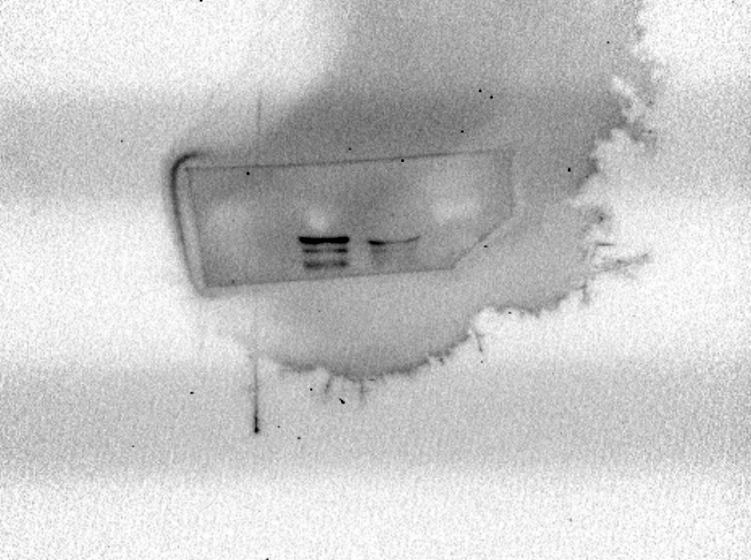


PLK1(67kDa)


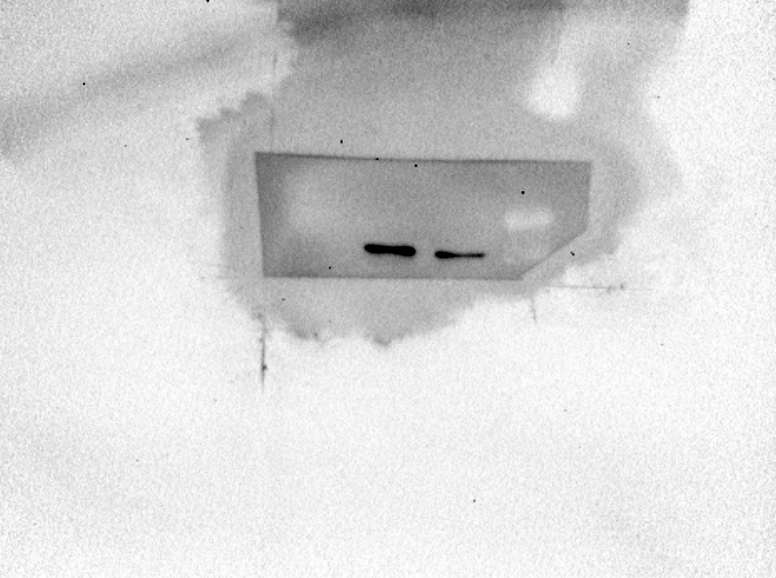


PAK2(58kDa)


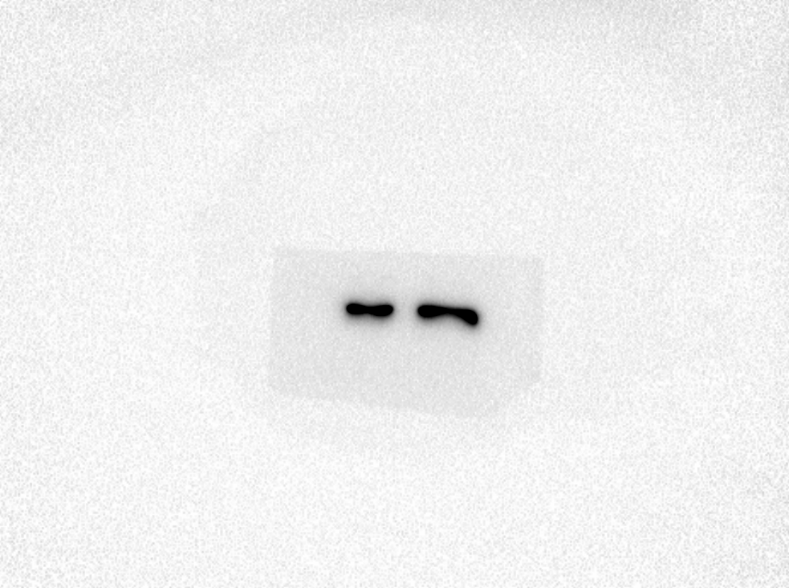


GAPDH (37kDa)

Fig. 5. PAK2 is associated with centrosomal protein PLK1. (B) PLK1 protein levels in control and Pak2-KD oocytes.

Input IgG PAK2 Input IgG PAK2


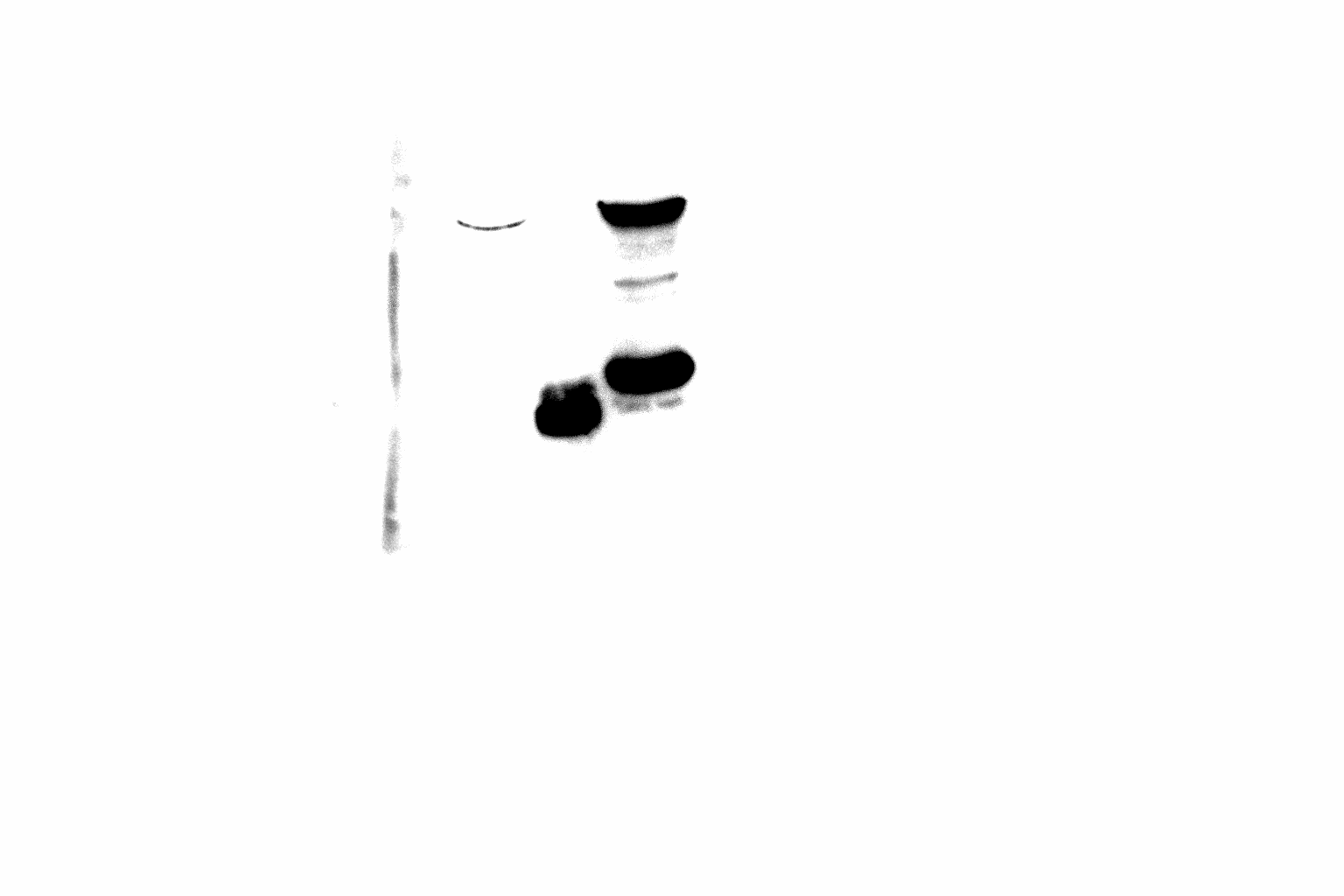

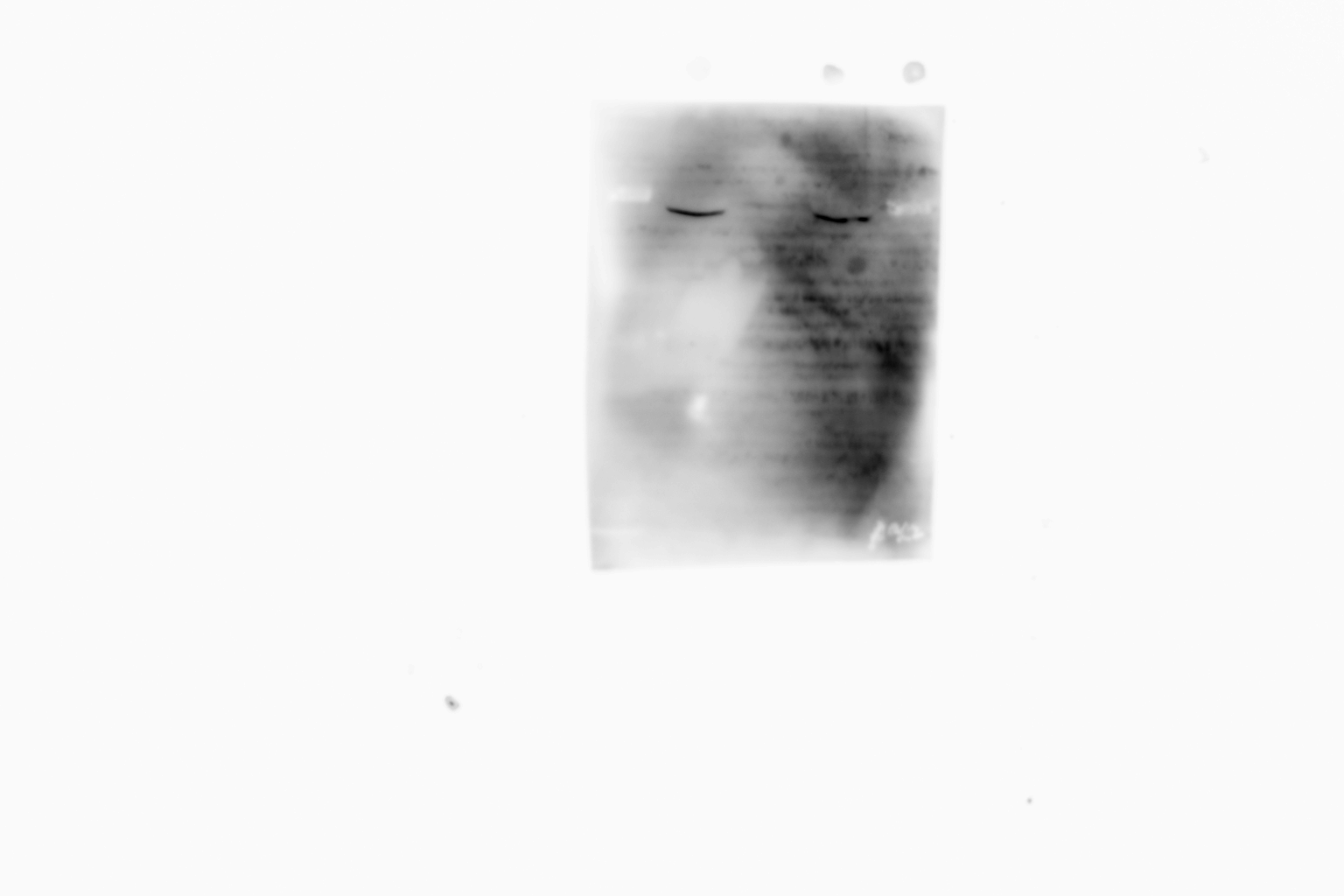


PLK1(67kDa)

PAK2 (58kDa)

Fig. 5. PAK2 is associated with centrosomal protein PLK1. (D) Co- IP was performed to determine the interaction between PAK2 and PLK1. Oocytes lysates was incubated with IgG and anti-PAK2 antibody, followed by incubation with protein G beads. The blots of IP eluates were probed with anti-PAK2 and anti-PLK1 antibodies, respectively.

Input IgG PLK1 Input IgG PLK1


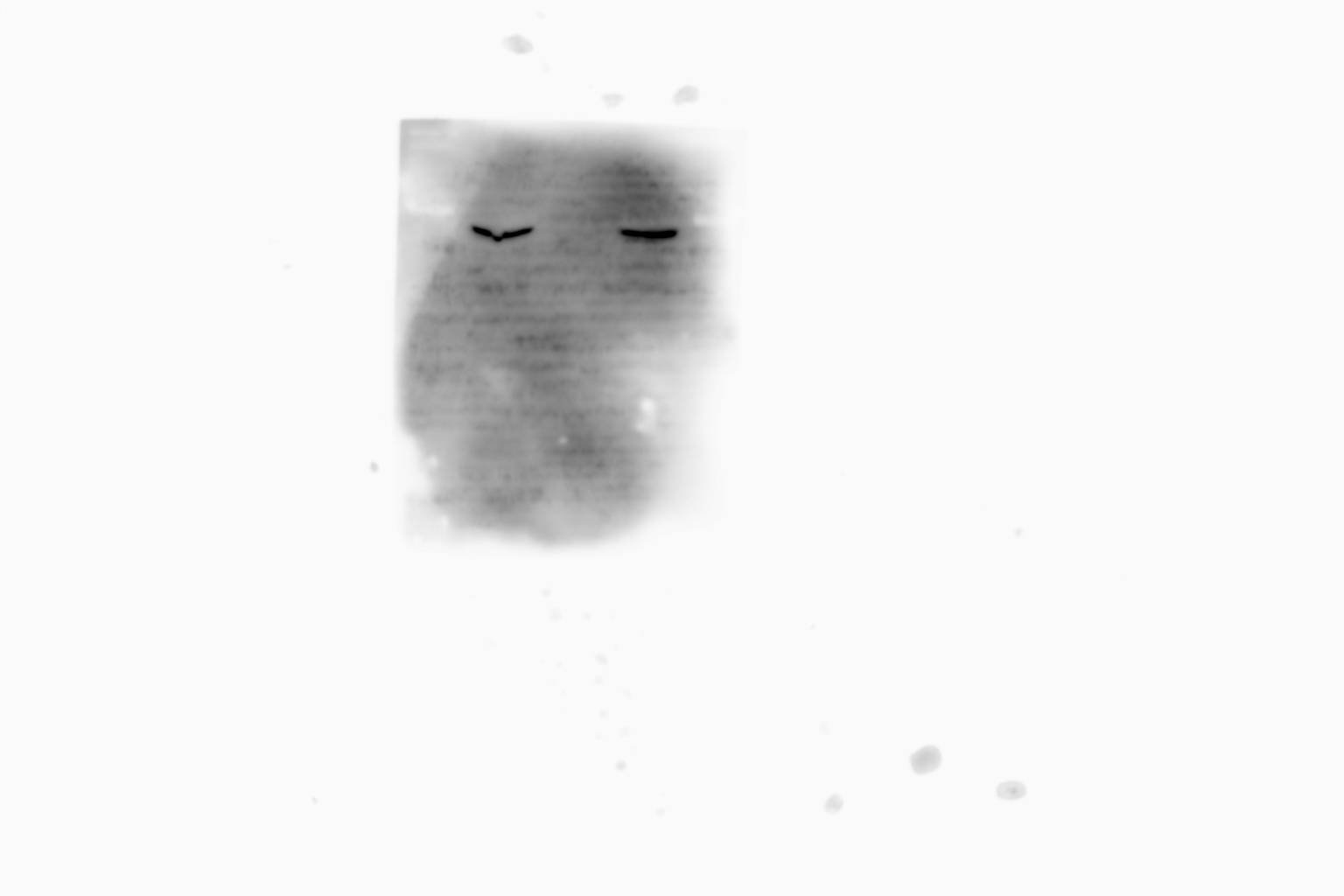




PAK2 (58kDa) PLK1(67kDa)

Fig. 5. PAK2 is associated with centrosomal protein PLK1. (E) Reciprocal Co-IP was performed with IgG and anti-PLK1 antibody. The blots of IP eluates were probed with anti-PLK1 and anti-PAK2 antibodies, respectively.
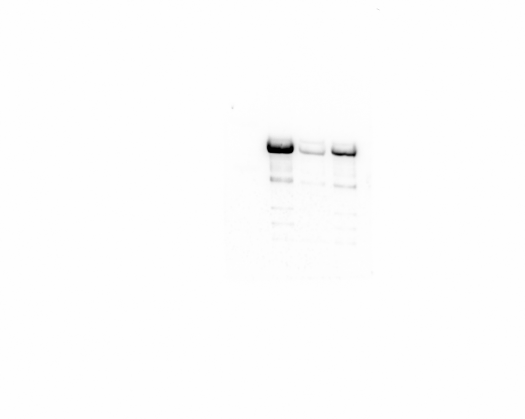
 PLK1(67kDa)


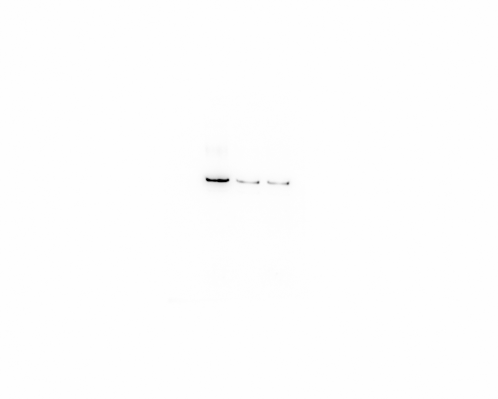


PAK2(58kDa)


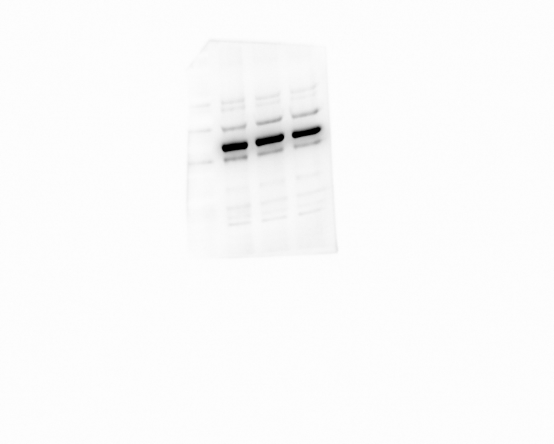


Tubulin (55kDa)

Fig. 6. PAK2 protects PLK1 from APC/C^Cdh1^-mediated degradation. (A) PLK1 protein levels in control, Pak2-KD, and Pak2-KD + MG132 oocytes. The blots were probed with PAK2, PLK1 and Tubulin antibodies.


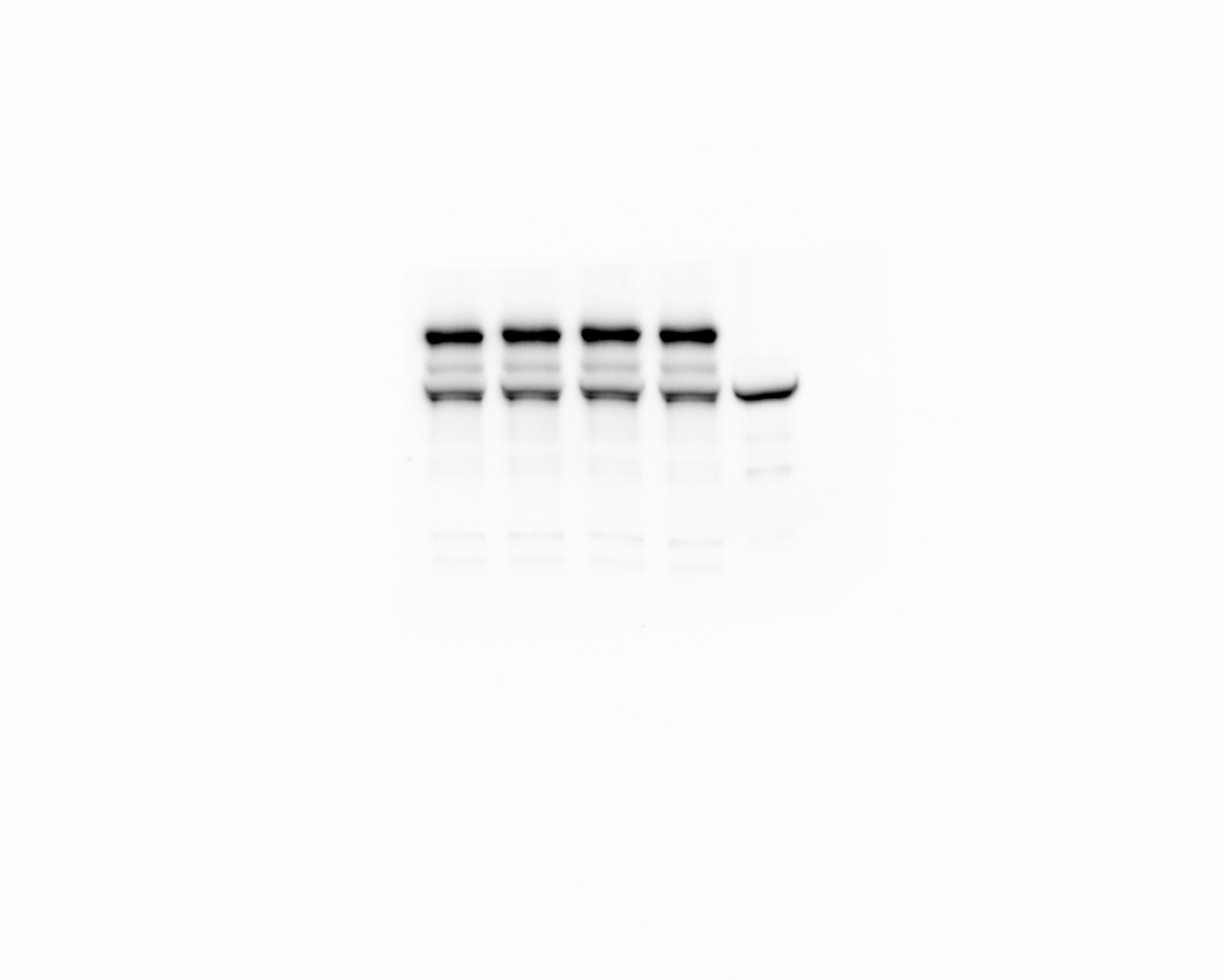


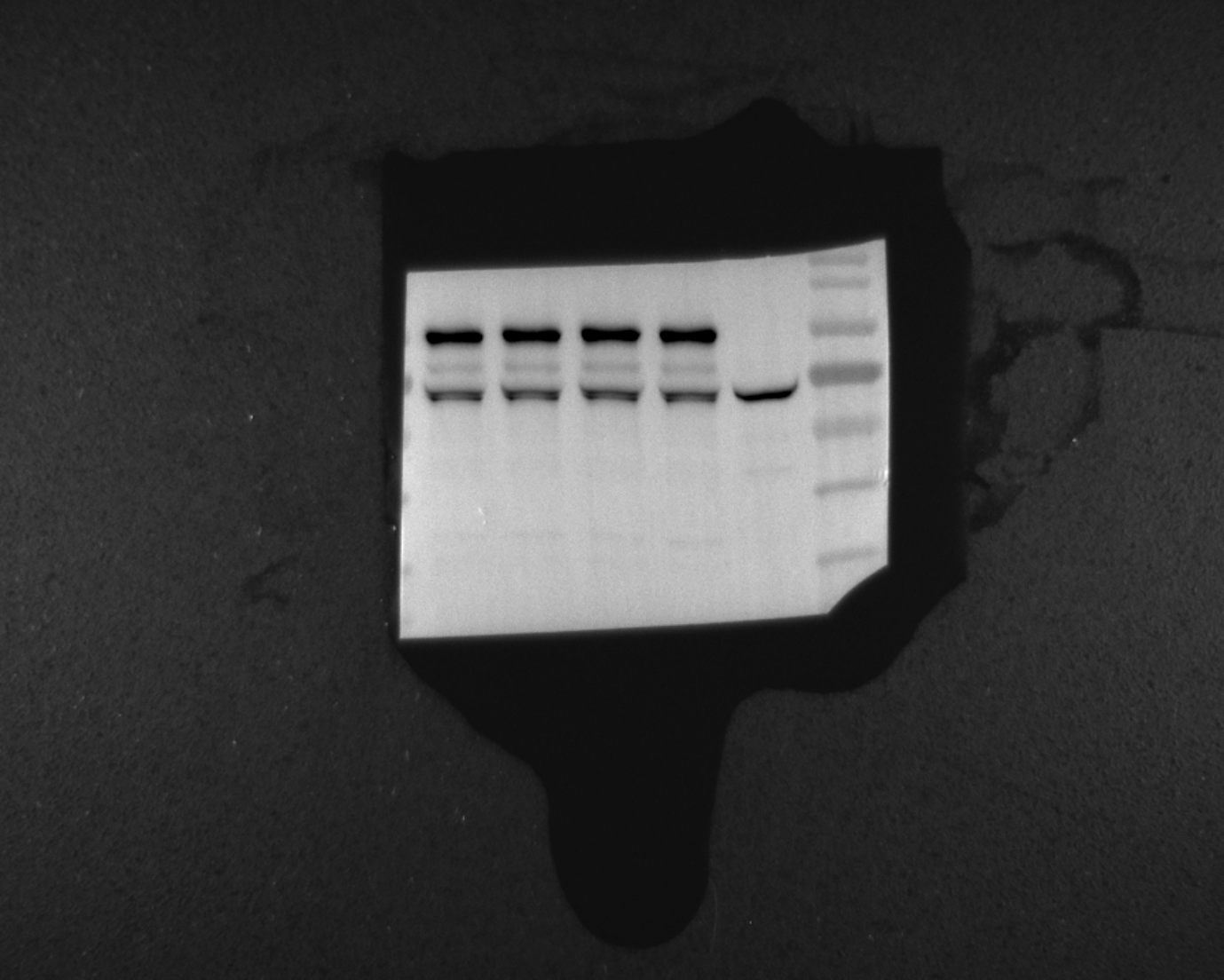


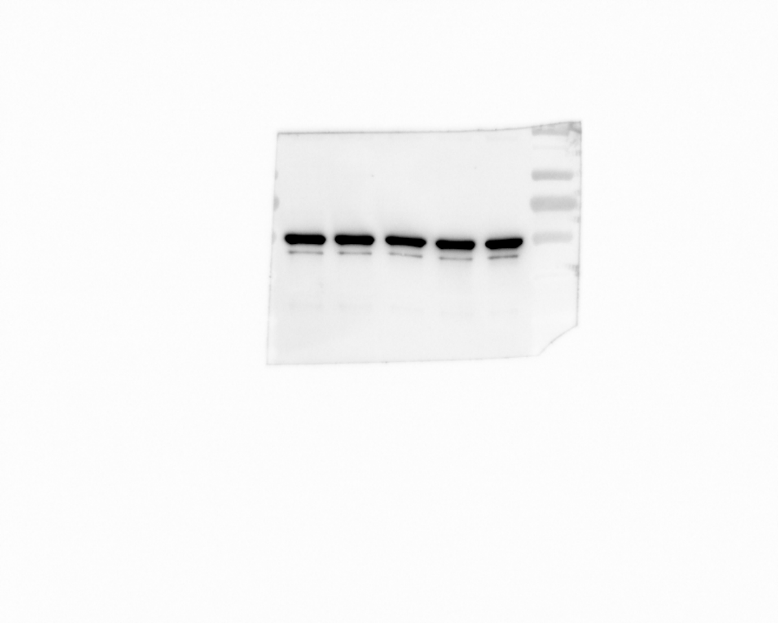


Myc-PLK1

PLK1(67kDa)

Tubulin(55kDa)

Fig. 6. PAK2 protects PLK1 from APC/C^Cdh1^-mediated degradation. (B) Western blots show that the various mutant PLK1 proteins were expressed to a similar extent. The blots were probed with PLK1 and Tubulin antibodies.


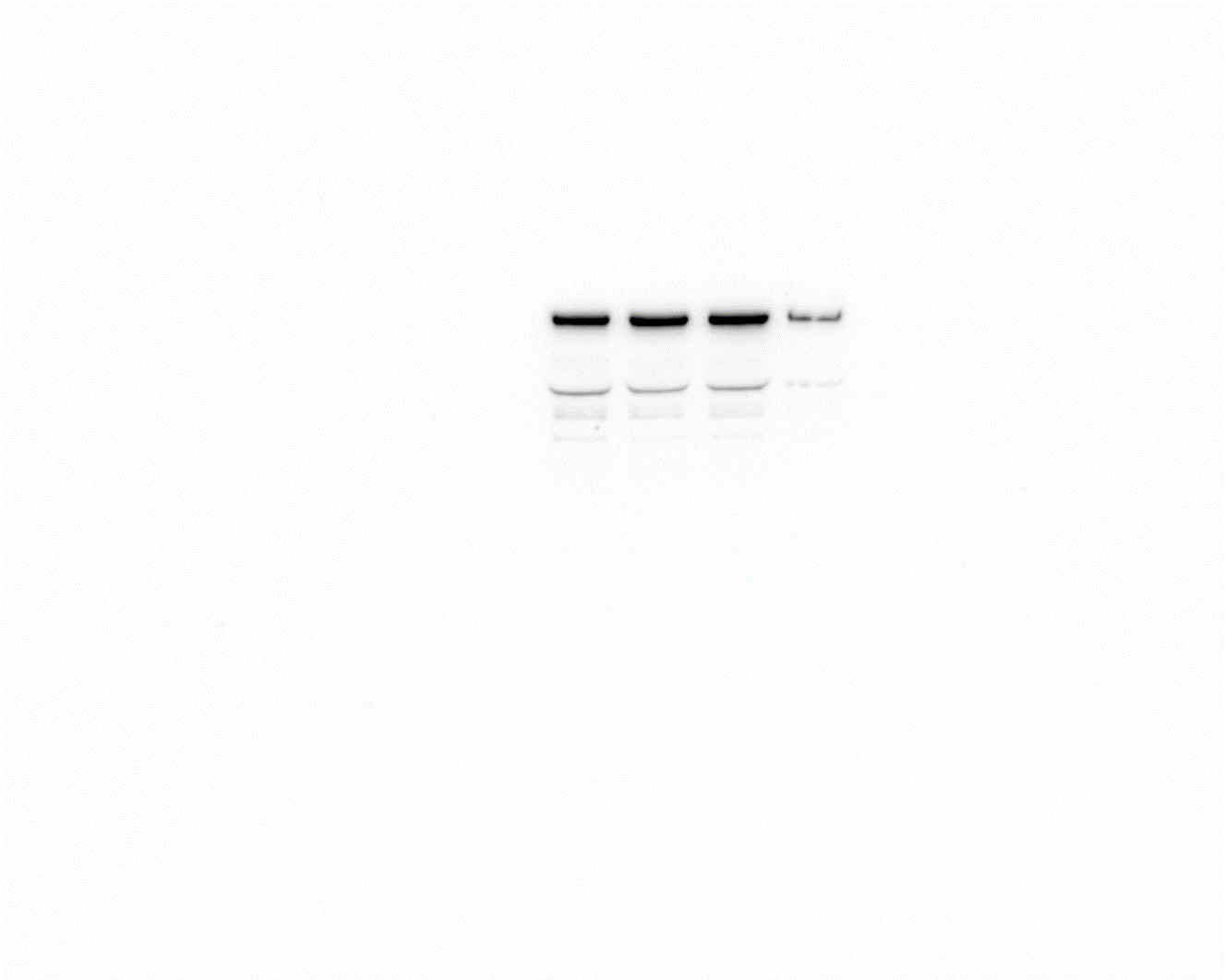
Myc-PLK1


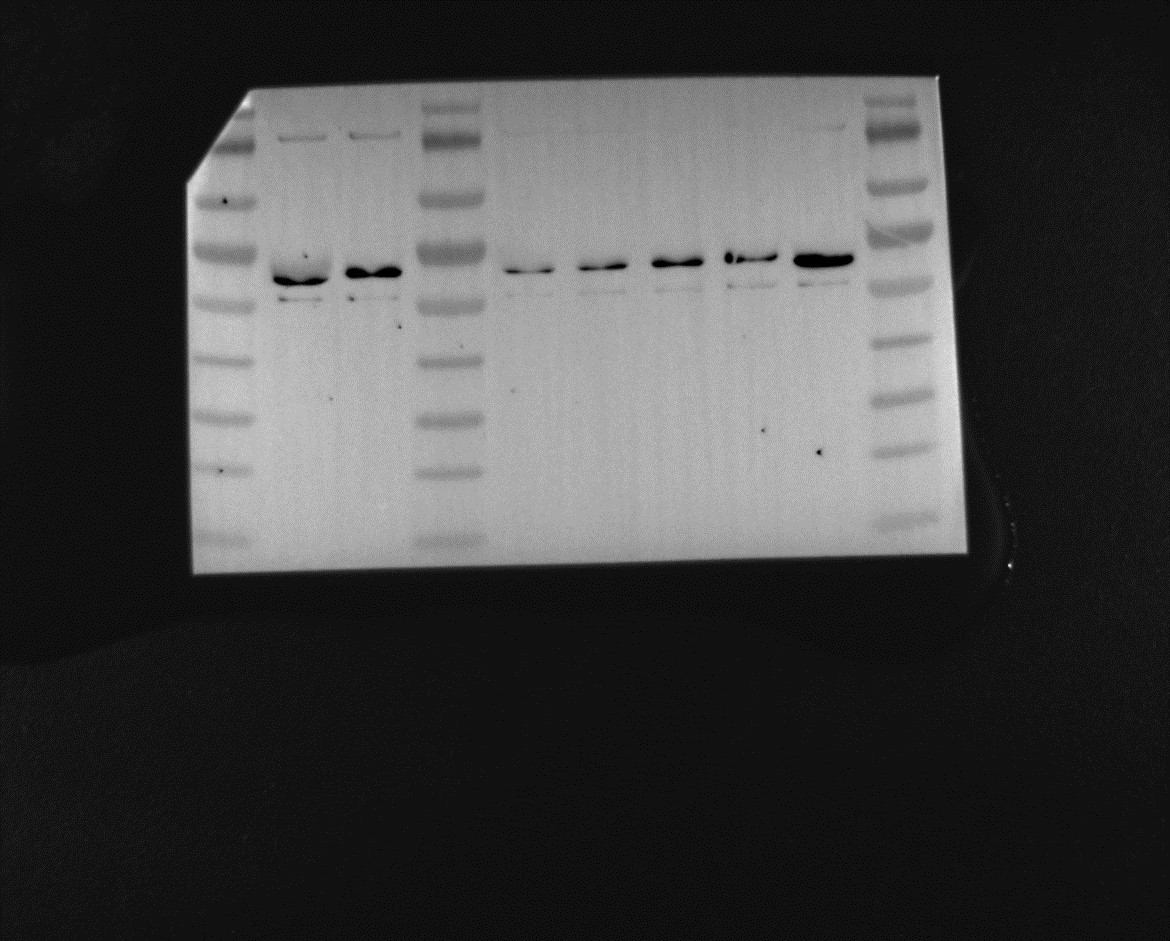


PAK2(58kDa)


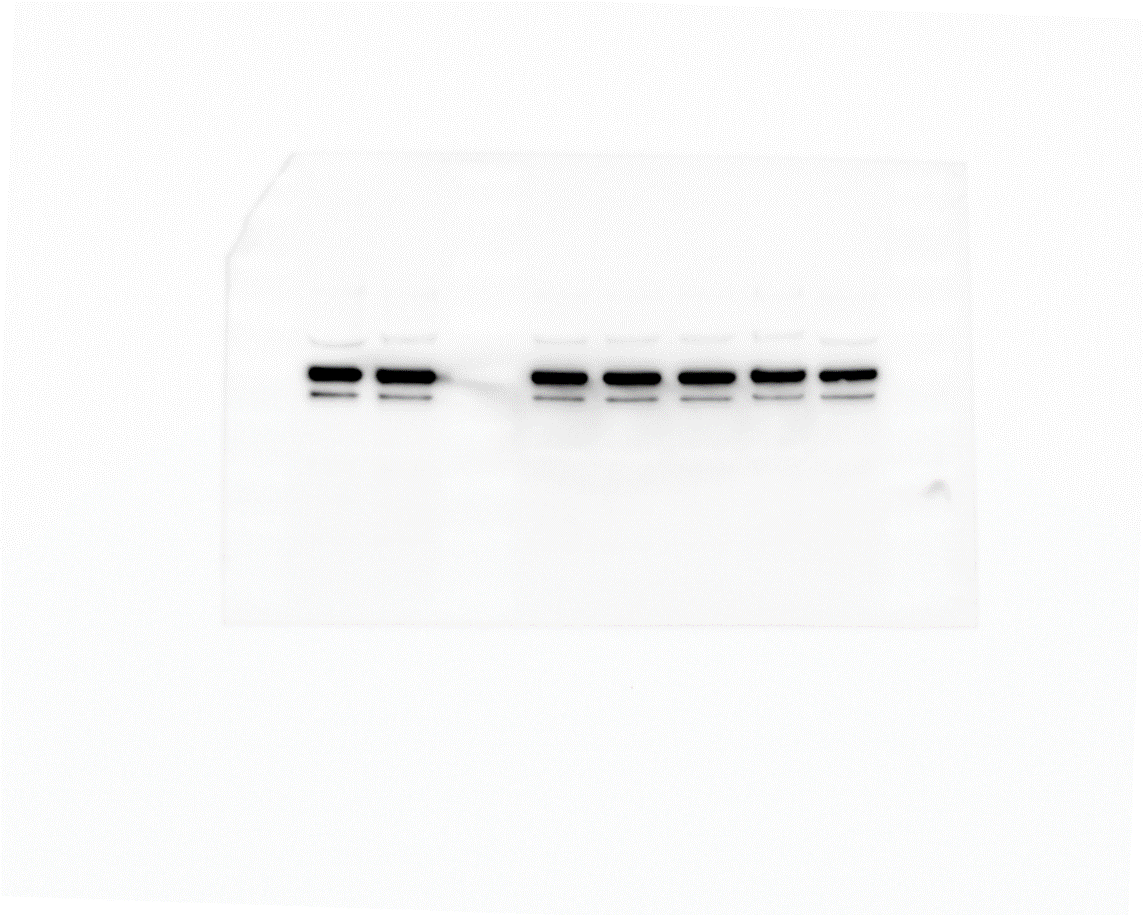


Tubulin(55kDa)

Fig. 6. PAK2 protects PLK1 from APC/C^Cdh1^-mediated degradation. (C) PLK1 protein levels in control, *Pak2*-KD + *Plk1*-L340A, *Pak2*-KD + *Plk1*-R337A, *Pak2*-KD + T210D and *Pak2*-KD + *Plk1*-WT oocytes. The blots were probed with Myc, PAK2 and Tubulin antibodies.


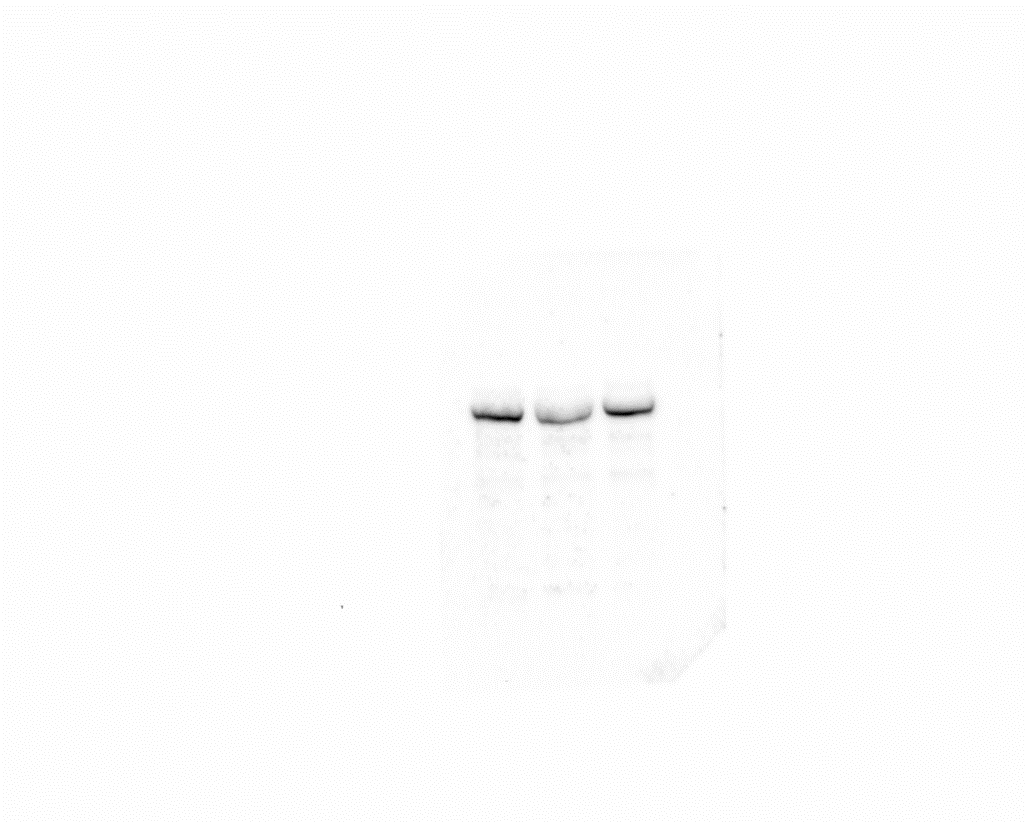


PLK1(67kDa)


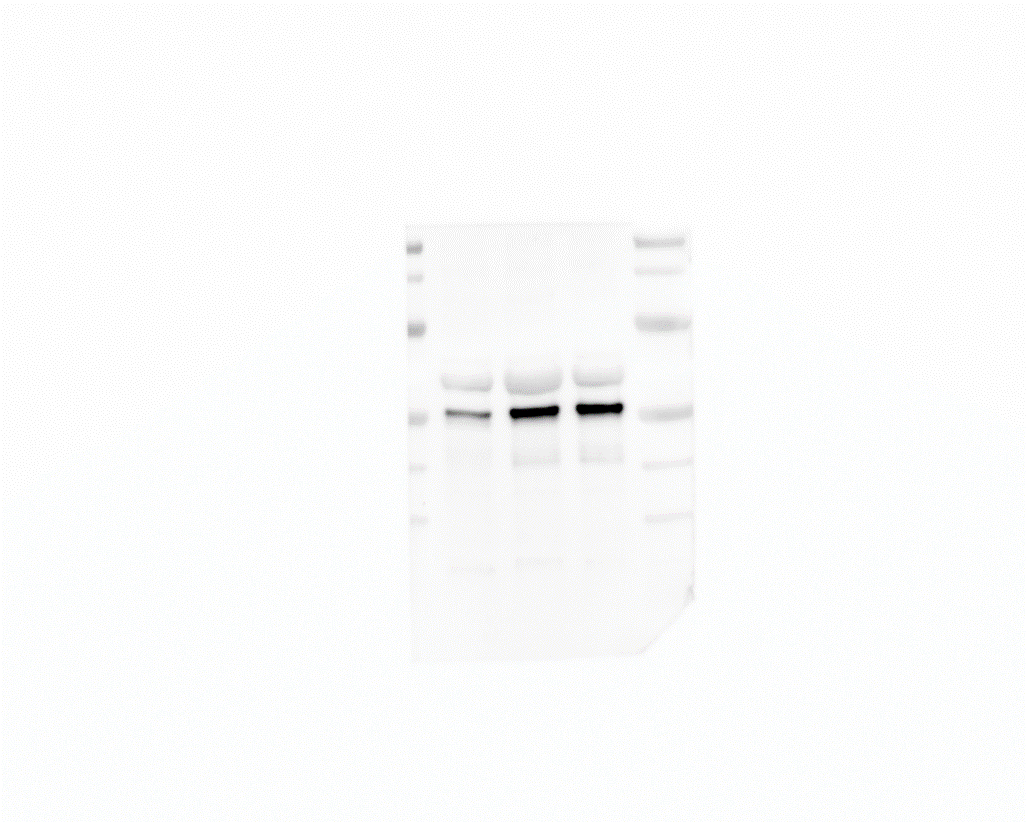


Cdh1(55 kDa)


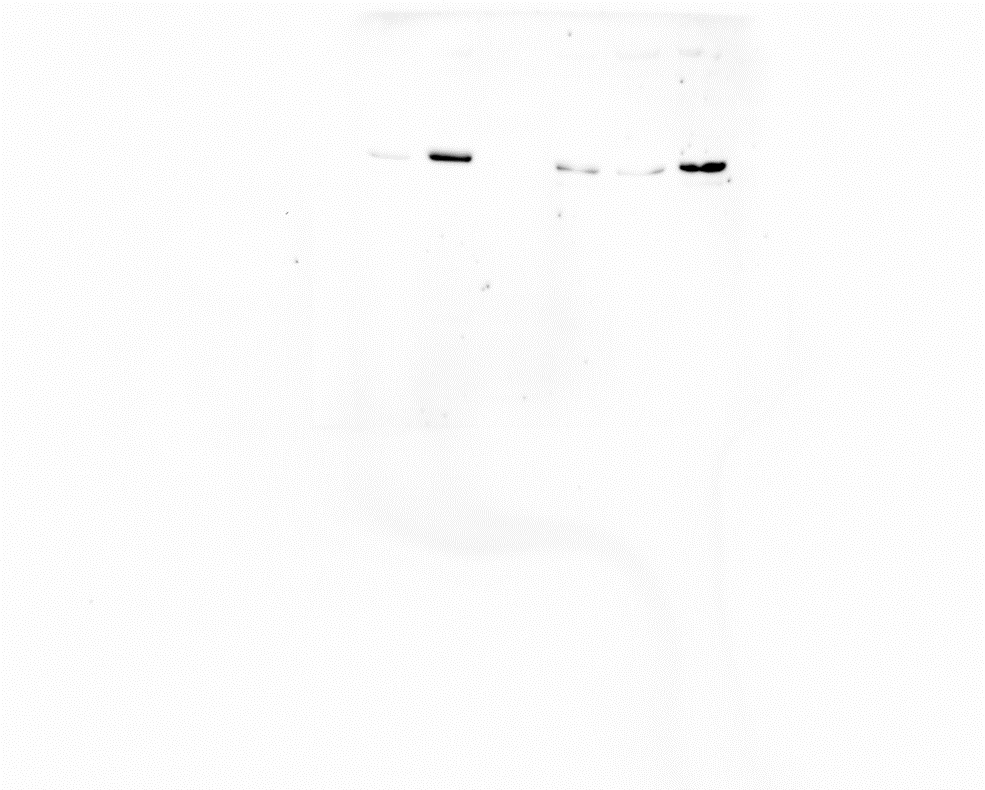


PAK2 (58kDa)


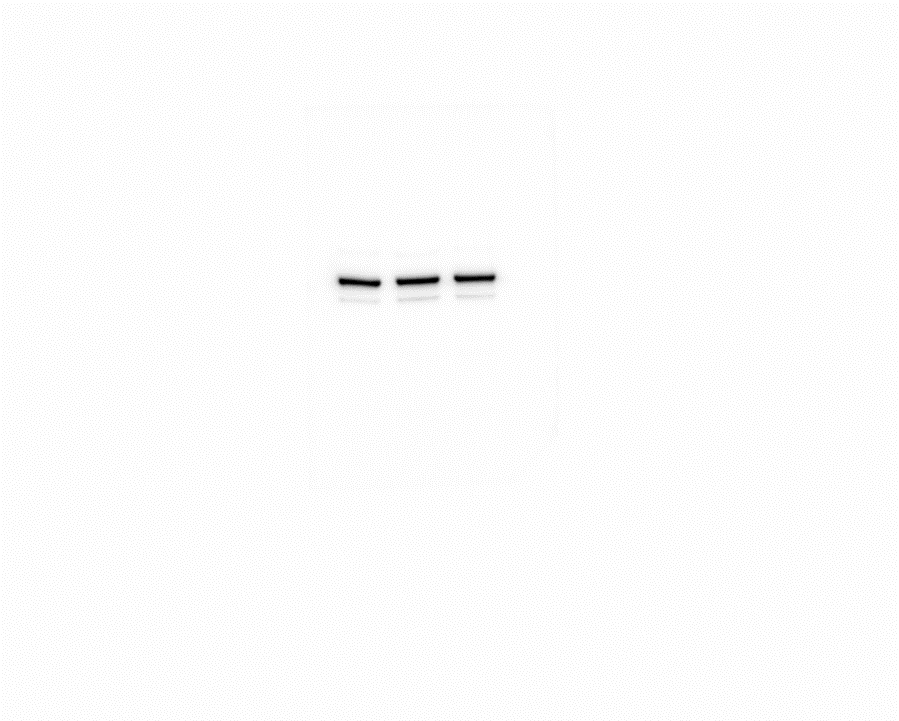


Tubulin(55kDa)

Fig. 6. PAK2 protects PLK1 from APC/C^Cdh1^-mediated degradation. (D) PLK1 protein levels in control, *Pak2*-KD, and *Pak2*-KD + *Cdh1*-KD oocytes. The blots were probed with PAK2, Cdh1, PLK1 and tubulin antibodies.


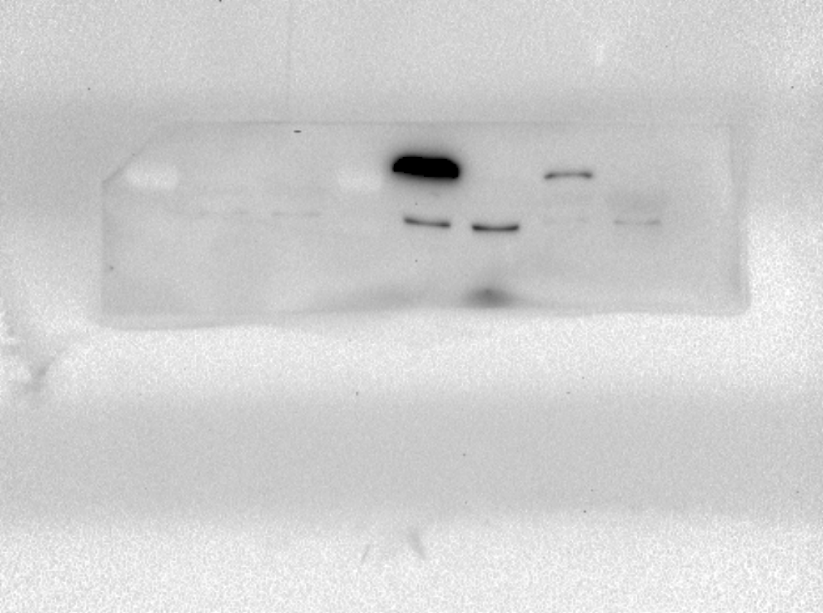


Endogenous

PAK2(58kDa) Exogenous


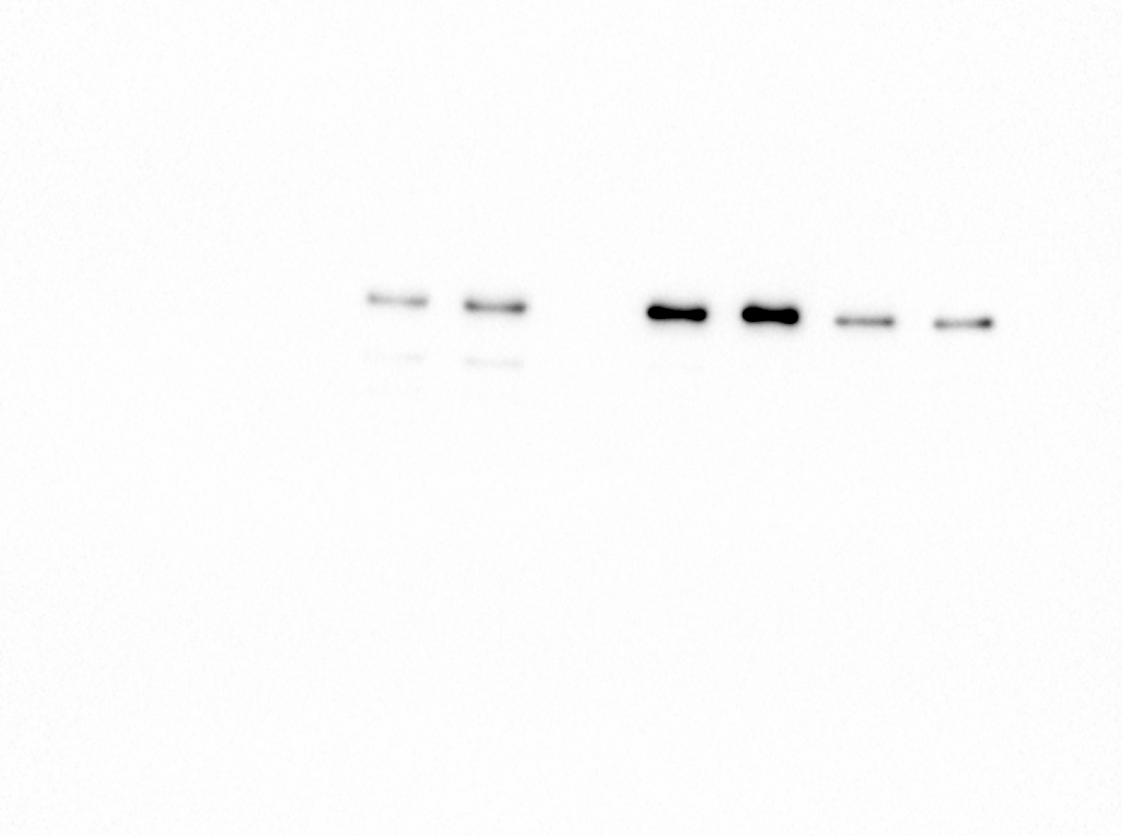


GAPDH (37kDa)

Fig. S1. Localization of Myc-PAK2 in mouse oocytes. (A) Representative western blot results show efficient overexpression of exogenous PAK2 protein.
